# Sleep and breast and prostate cancer risk in the MCC-Spain study

**DOI:** 10.1038/s41598-022-25789-9

**Published:** 2022-12-16

**Authors:** Michelle C. Turner, Esther Gracia-Lavedan, Kyriaki Papantoniou, Nuria Aragonés, Gemma Castaño-Vinyals, Trinidad Dierssen-Sotos, Pilar Amiano, Eva Ardanaz, Alba Marcos-Delgado, Ana Molina-Barceló, Juan Alguacil, Yolanda Benavente, Thalia Belmonte, José J. Jiménez-Moleón, Rafael Marcos-Gragera, Beatriz Pérez, Inés Gómez-Acebo, Marina Pollán, Manolis Kogevinas

**Affiliations:** 1 Barcelona Institute for Global Health (ISGlobal), Doctor Aiguader, 88, 08003, Barcelona, Spain; 2 Universitat Pompeu Fabra (UPF), Barcelona, Spain; 3 CIBER Epidemiología y Salud Pública (CIBERESP), Madrid, Spain; 4 Department of Epidemiology, Center of Public Health, Medical University of Vienna, Vienna, Austria; 5 Epidemiology Section, Public Health Division, Department of Health of Madrid, Madrid, Spain; 6 IMIM (Hospital del Mar Medical Research Institute), Barcelona, Spain; 7 Faculty of Medicine, University of Cantabria, Santander, Spain; 8 IDIVAL, Santander, Spain; 9 Ministry of Health of the Basque Government, Sub Directorate for Public Health and Addictions of Gipuzkoa, San Sebastian, Spain; 10 Biodonostia Health Research Institute, Epidemiology of Chronic and Communicable Diseases Group, San Sebastian, Spain; 11 Navarre Public Health Institute, Pamplona, Spain; 12 Instituto de Biomedicina, Universidad de León, León, Spain; 13 Cancer and Public Health Area, FISABIO-Public Health, Valencia, Spain; 14 Centro de Investigación en Recursos Naturales, Salud y Medio Ambiente (RENSMA), Universidad de Huelva, Campus Universitario de El Carmen, Huelva, Spain; 15 Cancer Epidemiology Research Programme, IDIBELL Institut Català d’Oncologia L’Hospitalet de Llobregat, Barcelona, Spain; 16 IUOPA, University of Oviedo and ISPA (Health Research Institute of Asturias), Oviedo, Spain; 17 Instituto de Investigación Biosanitaria ibs.GRANADA, Granada, Spain; 18 Departamento de Medicina Preventiva y Salud Pública, Universidad de Granada, Granada, Spain; 19 Research Group on Statistics, Econometrics and Health (GRECS), University of Girona, Girona, Spain; 20 Genetic Descriptive, Genetic and Prevention Epidemiology Group, Biomedical Research Institute of Girona (IDIBGI), Girona, Spain; 21 School of Medicine, University of Girona (UdG), Girona, Spain; 22 Epidemiology Unit and Girona Cancer Registry, Oncology Coordination Plan, Department of Health Government of Catalonia, Catalan Institute of Oncology, Girona, Spain; 23 National Center for Epidemiology, Instituto de Salud Carlos III, Madrid, Spain

**Keywords:** Cancer, Epidemiology, Risk factors

## Abstract

Breast and prostate cancers have been associated with circadian disruption. Some previous studies examined associations of sleep duration and breast or prostate cancer risk though findings remain inconsistent. This study examines associations of a range of detailed sleep characteristics and breast and prostate cancer risk in a large-scale population-based case–control study, MCC-Spain. A total of 1738 incident breast cancer cases, 1112 prostate cancer cases and frequency matched controls (n = 1910, and 1493 respectively) were recruited. Detailed data on habitual sleep duration, quality, timing, and daytime napping (“siesta”) were collected at recruitment. Additional data on sleep habits during both the previous year and at age 40 years were also subsequently captured. Adjusted odds ratios (ORs) and 95% confidence intervals (CI) were estimated. There were no associations of habitual sleep duration (h), timing of sleep, or any or specific sleep problems, and either breast and prostate cancer risk. There was a significant positive association of ever taking habitual siestas at recruitment and breast cancer risk (OR = 1.22, 95% CI 1.06–1.42), which strengthened with increased frequency or duration. There were also significant positive associations observed for both breast and prostate cancer, among those reporting recent sleep problems, but not sleep problems at age 40 years, in a subsequent circadian questionnaire. Adverse associations with siesta and disturbed sleep during the previous year likely reflect symptoms of developing/diagnosed cancer and comorbidities. Overall, there was no clear association between various sleep characteristics and breast or prostate cancer risk observed.

## Introduction

There is growing public health concern surrounding insufficient sleep with increasing proportions of the adult population reporting sleeping less than the recommended 7–8 h per night^1^. In addition, poor sleep quality and insomnia symptoms affect large proportions of adults^2^. Insufficient sleep has been associated with a range of adverse physical and mental health outcomes including various neurological, cardiovascular and metabolic, reproductive, and immune-related effects^1^. There are also concerns surrounding long sleep duration and adverse health^1,3,4^. Recent meta-analyses of sleep duration and total mortality reported U-shaped associations, with increased risks of mortality for those sleeping either less than, or greater than 7 h/day^3,4^.

Some epidemiological studies have examined associations of sleep duration and cancer risk, including breast and prostate cancer. Breast and prostate cancers are among the most commonly diagnosed cancers, and leading causes of cancer death worldwide, share some common hormonal and etiological features, and have been associated with circadian disruption^5,6^. Previous studies are generally inconsistent and have reported differing associations of both short^7–11^ and long^10,12,13^ sleep duration, and breast or prostate cancer risk^14–17^. Recent meta-analyses have reported no clear evidence of associations of either short or long sleep duration and breast or prostate cancer risk^18–20^.

Some other studies have examined different indices of sleep problems and breast or prostate cancer risk. Some adverse associations of poor sleep quality, disturbed sleep, inappropriate timing of sleep, or sleep disorders were observed^21–26^. In one study, men reporting greater than 1 h of regular social jetlag (difference in waking time between weekdays and weekend) had a significantly increased risk of incident prostate cancer^27^. There is also a growing literature on obstructive sleep apnea and breast and prostate cancer risk^28–30^.

Limitations of previous studies include that they typically have had limited data to characterise sleep and have often considered few measures of sleep, usually with a single question for a single point in time^31,32^. Additional large-scale, population-based studies, with detailed data on different dimensions of sleep (e.g. duration, quality, timing) are needed to better understand potential associations with both breast and prostate cancer risk^31,32^. This study seeks to examine associations of a range of detailed personal sleep characteristics, including sleep duration, sleep quality, timing of sleep, and daytime napping (“siesta”), and other relevant circadian data, and both breast and prostate cancer risk in the large-scale MCC-Spain study.

## Methods

### Study population

MCC-Spain is a population-based case–control study of five common cancers (breast, prostate, colorectal, gastro-oesophageal, chronic lymphocytic leukemia) conducted from 2008 to 2013. The study methods and objectives are described elsewhere^33^. Incident cases of invasive and non-invasive breast cancer (International Classification of Diseases 10 (ICD-10) C50, D05.1, D05.7) identified in all public reference hospitals in 10 regions of Spain (Asturias, Barcelona, Cantabria, Girona, Guipuzcoa, Huelva, Leon, Madrid, Navarra, and Valencia) and prostate cancer (ICD-10 C61, D07.5) in seven regions (Asturias, Barcelona, Cantabria, Granada, Huelva, Madrid, and Valencia) were recruited as rapidly as possible following diagnosis^34^. For inclusion in the study, cases were aged from 20 to 85 years, confirmed histologically, and had lived for at least 6 months in the study area prior to diagnosis. Exclusion criteria included having either a communication difficulty or a prohibitive physical condition. A total of 1738 female breast cancer cases and 1112 prostate cancer cases were recruited into the study. Responses rates for breast and prostate cancer cases varied by study centre and were 71% and 72% overall respectively. A variety of clinical data were obtained from medical records.

Controls were randomly selected from the rosters of general practitioners from public primary health centres located within the included hospital catchment areas and were frequency-matched to the entire distribution of all cancer cases included in the MCC-Spain study by sex and age (5-year groups). The same control set was used for all five cancer cases in the study. A total of 1910 eligible female controls and 1493 eligible male controls were recruited among the relevant regions here, with response rates of 52% and 56% overall respectively, among those contacted. The study was reviewed and approved by the ethics committees of all participating institutions. The study was conducted in accordance with the ethical standards of each research committee, and with the 1964 Helsinki Declaration and its later amendments or comparable ethical standards. The protocol of MCC-Spain was approved by each of the ethics committees of the participating institutions (Ethical Committee of Clinical Research of Barcelona, Cantabria, Girona, Granada, Gipuzkoa, Huelva, León, Principado de Asturias, Madrid, Navarra and Valencia). The database was registered in the Spanish Agency for Data Protection, number 2102672171. Study participants provided written informed consent prior to enrolment.

### Data collection

Detailed data were collected in face-to-face interviews at recruitment by trained personnel on a range of habitual nighttime and daytime sleep-related characteristics in the main study questionnaire including: the average number of hours slept per night; if the participant has ever experienced a long period (at least 1 year) with sleeping problems and if so, the type of sleeping problems (problems falling asleep, waking up in the middle of the night, taking medication to fall asleep, other) and the age at which the sleeping problems began and ended; if the participant has ever experienced a long period (at least 1 year) with frequent changes in the time they usually go to sleep, and if so, the reason for the frequent changes (night shift work or other); and the number of days per week and average duration (min) of daytime napping (“siesta”). For participants reporting going to sleep at approximately the same time during the past 10 years, the time the participant usually goes to sleep was captured. Interviews with cancer cases were scheduled as shortly as possible following cancer diagnosis.

A subsequent supplementary questionnaire on an expanded range of circadian-related topics was also later administered by telephone, with all breast and prostate cancer cases and controls willing to be re-contacted invited to participate (68.2% and 70.0% of breast cancer cases and controls, and 74.5% and 71.9% of prostate cancer cases and controls respectively), collecting further retrospective information on a range of personal sleep habits experienced either during the (1) previous year or (2) at age 40 years including: the number of nights per week and number of times per night the participant wakes up during sleep (and if the reason for waking up is to go to the bathroom); self-reported level of rest during sleep (from 1–10); the time the participant usually turns off the lights to go to sleep and the time the participant usually wakes up, both during work days and days off (at age 40 years only). Information on chronotype was also captured (Munich Chronotype Questionnaire)^35^. The supplementary circadian questionnaire was administered a mean (SD) of 3.0 (0.9) and 3.1 (0.7) years following the main face-to-face recruitment interview among breast cancer and prostate cancer cases respectively and 3.0 (1.1) and 2.9 (0.9) years for controls.

Data on a range of sociodemographic and lifestyle factors were collected in the main study interview including age, education, socioeconomic status (score constructed using information on the participant education level (range 0–3) and occupation (range 0–2), and parental education level (range 0–2)), cigarette smoking status (1 year prior to interview), family history of breast and prostate cancer in first degree relatives, body mass index (BMI), physical activity level (metabolic equivalent (METs) h/week during the past 10 years), diet and alcohol consumption. Among women, additional hormonal and reproductive data were also collected, including parity, age at first child, oral contraceptive use, hormone replacement therapy use, age at menarche, and menopausal status. Occupational history data were obtained for all jobs held for more than 1 year. Data collected included job title, tasks, start and stop dates, and information on shifts worked (time schedule, hours per day, percentage of time worked in the morning, evening or night). Ever night shift workers were defined as those having worked either partly or entirely between the hours of 24:00 to 06:00 at least three nights per month^36,37^.

### Statistical analysis

Adjusted odds ratios (ORs) and 95% confidence intervals (CIs) for associations of various personal habitual sleep characteristics captured at recruitment in the main study questionnaire including sleep duration, sleep quality, timing of sleep, and siesta and incident breast or prostate cancer risk were estimated using multivariable unconditional logistic regression models. Generalized additive models were used to examine the shape of associations of sleep duration and breast and prostate cancer risk. Sensitivity analyses were conducted with mutual adjustment for other sleep characteristics as well as with exclusion of those reporting any sleep problem in the past 5 years. Potential effect modification of associations of sleep characteristics by other sleep variables (sleep duration (< 7 h vs 7 vs ≥ 8 h), ever sleep problems, timing of sleep (< 00 h vs ≥ 00 h), ever siesta) was assessed with two-sided *p* values assessed according to the likelihood ratio statistic. Potential effect modification of associations of sleep characteristics by other personal factors including age group (< 60 years vs ≥ 60 years), BMI (< 25 kg/m^2^ vs ≥ 25 kg/m^2^), menopausal status (pre/peri vs post), night shift work history (never, ever, housewife), chronotype (morning, neither, evening), and residence outdoor blue light spectrum (participants in Barcelona and Madrid only) (< median vs ≥ median) was also examined^38^. Analyses were conducted according to disease subphenotypes (receptors for breast cancer) or aggressiveness of disease (Gleason score for prostate cancer) using multinomial logistic regression models. Finally, associations of additional sleep characteristics captured using the supplementary circadian questionnaire, including the number of nights per week and number of times per night the participant wakes up during sleep (and if the reason for waking up is to go to the bathroom), self-reported level of rest during sleep, sleep duration and timing of sleep on both weekdays and weekends, and social jetlag, during both the previous year and at age 40 years were also estimated in a subset of participants. Statistical analysis was conducted using R version 3.5^39^.

### Ethics approval and consent to participate

The study was reviewed and approved by the ethics committees of all participating institutions. Study participants provided written informed consent prior to enrolment.

## Results

A total of 1581 breast cancer cases and 1609 controls were retained for analysis following exclusion of participants with missing data on habitual sleep variables, key covariates, or had reported a previous personal history of cancer. Similarly, a total of 1013 prostate cancer cases and 1179 controls were retained. Table 1 presents the distribution of selected participant characteristics among breast and prostate cancer cases and control participants. Breast cancer cases had a slightly lower mean (SD) age (56.0 (12.5) years) than controls (58.3 (13.3)). Madrid, Barcelona, and Leon contributed the largest number of breast cancer cases and Barcelona, Madrid, and Cantabria the largest number of prostate cancer cases. Breast cancer cases were more likely to be of postmenopausal status and have a family history of breast cancer than controls. Prostate cancer cases had a somewhat lower level of educational attainment and socioeconomic score than controls, as well as a greater previous family history of prostate cancer.

**Table 1:** Distribution of participant characteristics, breast and prostate cancer cases and controls, MCC-Spain, 2008–2013.

	Breast cancer cases n = 1581 %	Breast cancer controls n = 1609 %	*p* value	Prostate cancer cases n = 1013 %	Prostate cancer controls n = 1179 %	*p* value
Age (years), mean (SD)	56.0 (12.5)	58.3 (13.3)	< 0.001	65.9 (7.3)	66.4 (8.3)	0.10
**Centre**			< 0.001			< 0.001
Asturias	4.2%	7.1%		1.6%	7.3%	
Barcelona	16.6%	14.4%		35.0%	30.1%	
Cantabria	8.3%	11.2%		15.6%	14.2%	
Girona	2.9%	3.4%		–	–	
Granada	–	–		5.9%	9.6%	
Guipuzcoa	12.1%	12.9%		–	–	
Huelva	5.6%	4.2%		4.6%	7.0%	
Leon	13.8%	11.6%		–	–	
Madrid	19.9%	21.0%		29.5%	25.6%	
Navarra	13.0%	10.1%		–	–	
Valencia	3.7%	4.0%		7.7%	6.2%	
**Education**			0.16			< 0.001
< Primary	14.6%	16.6%		23.2%	18.9%	
Primary	32.9%	31.6%		39.5%	34.9%	
Secondary	32.7%	30.3%		21.8%	27.6%	
University	19.8%	21.6%		15.5%	18.7%	
**Socioeconomic score**			0.21			0.005
Low (0–2)	30.9%	31.9%		42.6%	36.6%	
Medium (3–5)	52.9%	49.0%		47.2%	47.4%	
High (6–7)	16.1%	17.3%		10.2%	13.7%	
**Cigarette smoking**			0.05			0.67
Never	55.2%	59.2%		29.0%	27.5%	
Former	19.7%	19.4%		47.7%	49.4%	
Current	24.5%	21.3%		22.8%	22.8%	
**Family history of breast/prostate cancer**			< 0.001			< 0.001
None	82.3%	87.8%		80.2%	88.2%	
Any	15.1%	8.6%		15.9%	6.1%	
**BMI (kg/m**^**2**^**)**			0.41			0.69
< 25	48.4%	50.2%		25.9%	24.5%	
25–< 30	33.9%	31.7%		51.0%	51.1%	
30	17.7%	18.1%		23.1%	24.3%	
**Physical activity**			0.83			0.86
Inactive	41.9%	40.6%		40.4%	40.9%	
Slightly active	17.8%	18.3%		13.3%	12.3%	
Moderately active	12.7%	12.3%		11.8%	11.4%	
Very active	27.7%	28.8%		34.5%	35.5%	
**Alcohol consumption**^a^			0.41			0.35
< Median	42.7%	44.1%		42.0%	43.5%	
≥ Median	45.1%	43.6%		45.8%	43.4%	
**Parity**			0.07			
Nulliparous	20.6%	19.3%				
1–2 children	57.3%	55.1%				
3 + children	21.9%	25.3%				
**Age at first child**			0.69			
Nulliparous	20.6%	19.3%				
< 20 years	4.4%	4.0%				
20–24 years	23.3%	24.2%				
25–29 years	30.3%	32.0%				
30 + years	20.6%	19.9%				
**Age at menarche**			0.17			
< 12 years	20.2%	18.7%				
12–13 years	25.7%	23.4%				
14 + years	52.8%	55.4%				
**Oral contraceptive use**			0.83			
Never	52.0%	51.6%				
Ever	47.8%	48.3%				
**Hormone replacement therapy**			0.81			
Never	91.0%	89.6%				
Ever	6.8%	7.0%				
**Menopausal status**			0.002			
Premenopausal	63.8%	69.0%				
Postmenopausal	36.2%	31.0%				
**Night shift work**			< 0.001			0.07
Never	76.1%	71.0%		70.4%	72.3%	
Ever	13.0%	11.1%		29.4%	25.3%	
Housewife	8.7%	13.3%		-	-	
**Chronotype**			0.23			0.74
Morning	29.9%	32.5%		42.3%	39.7%	
Neither	31.6%	32.0%		27.7%	28.2%	
Evening	19.5%	17.7%		10.9%	10.3%	
**Outdoor blue light spectrum**^b^			0.003			< 0.001
< Median (0.15)	43.2%	32.6%		35.9%	47.5%	
≥ Median (0.15)	43.0%	48.4%		51.8%	47.3%	

Note in some cases the sum does not equal the total due to missing data.

^a^Median = 1.63 ethanol g/day in women and 18.8 ethanol grams/day in men.

^b^Participants in Barcelona and Madrid only, n = 491 breast cancer cases and n = 467 breast cancer controls, n = 574 prostate cancer cases and n = 623 controls, current address.

Supplemental Table 1 presents the distribution of selected participant characteristics among breast cancer controls by categories of sleep duration (h) and siesta. Some participant characteristics varied by categories of sleep duration including among women reporting sleeping < 6 h or 9 + h per night being of greater mean age, as well as having a lower level of education, a lower socioeconomic status, being a never smoker, having a higher BMI, a lower alcohol consumption, having a greater number of children, never using oral contraceptives, being of premenopausal status, and lifetime housewives. Women reporting ever taking siestas were of somewhat greater mean age, having greater alcohol consumption, and being nulliparous. Supplemental Table 2 presents the distribution of selected prostate cancer control characteristics by categories of sleep duration (h) and siesta. Some participant characteristics varied by categories of sleep duration with men reporting sleeping < 6 h or 9 + h per night also being of somewhat greater mean age, as well as having a lower level of education and socioeconomic status. Men reporting ever working night shifts reported a greater sleep duration. Men reporting ever taking siestas were more likely former or current smokers.

**Figure 1:**
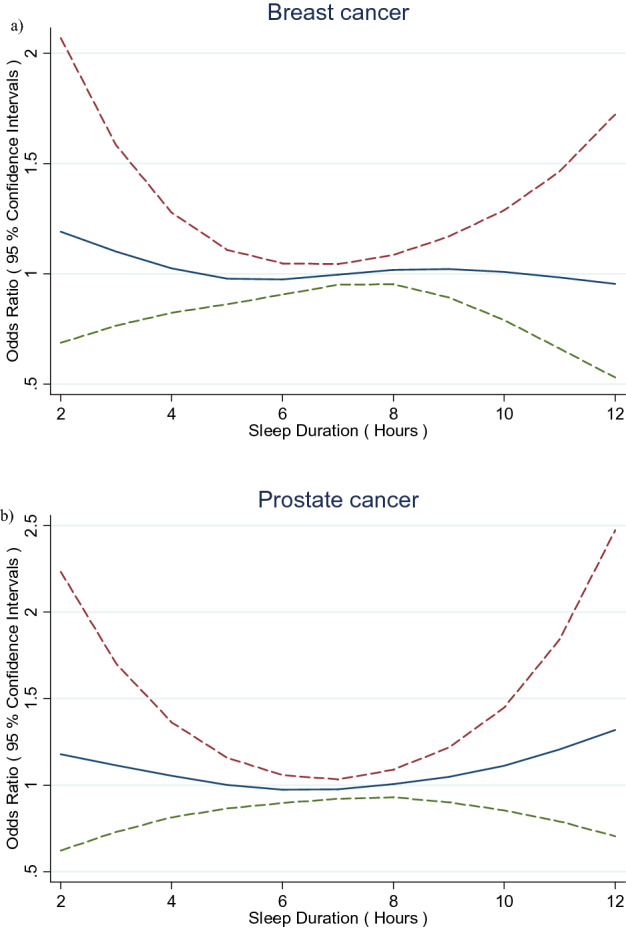
General additive models of associations of habitual sleep duration (h) and (**a**) breast and (**b**) prostate cancer risk, MCC-Spain, 2008–2013.

Associations of various habitual sleep characteristics captured at recruitment in the main study questionnaire and incident breast and prostate cancer risk are presented in Table 2. For breast cancer, there was no association with categories of sleep duration (h) (see also Fig. 1a). There was also no association among women reporting ever having a long period (at least 1 year) with sleep problems, or according to duration (years) of sleep problems, timing of sleep problems (last 5 years), or timing of sleep. There was no association among women reporting specific sleep problems or frequent changes in the time they go to sleep (Supplemental Table 3). There was however, a significant positive association among women reporting ever taking siestas (OR = 1.22, 95% CI 1.06–1.42), which strengthened among those reporting siestas of greater frequency (3–5 or 6–7 days per week) or duration (30–59 or 60 + min) (*p*ʼs for trend = 0.004 and 0.001 respectively).

**Table 2:** Associations of various habitual sleep characteristics and breast and prostate cancer risk, MCC-Spain, 2008–2013.

	Breast cancer cases n = 1543	Breast cancer controls n = 1560	OR^a^	LCI	UCI	Prostate cancer cases n = 1008	Prostate cancer controls n = 1150	OR^b^	LCI	UCI
**Sleep duration (h)**										
< 6	178	206	0.92	0.72	1.18	139	149	1.11	0.83	1.49
6	290	304	0.96	0.78	1.19	203	233	1.05	0.81	1.35
7	483	479	1.00	–	–	288	347	1.00	–	–
8	452	432	1.02	0.84	1.23	270	293	1.17	0.92	1.49
9 +	140	139	1.01	0.76	1.33	108	128	1.06	0.77	1.47
Per 1 h			1.01	0.95	1.06			1.01	0.95	1.08
**Ever sleep problems**										
No	938	891	1.00	–	–	758	840	1.00	–	–
Yes	620	669	0.95	0.82	1.11	250	310	0.92	0.75	1.12
**Duration of sleep problems (years)**										
None	923	891	1.00	–	–	758	840	1.00	–	–
< 10	309	315	0.95	0.79	1.15	106	122	0.96	0.72	1.29
10–19	105	120	0.93	0.69	1.24	38	50	0.94	0.59	1.48
20 +	76	82	1.12	0.79	1.58	32	47	0.97	0.60	1.56
**Ever sleep problems in past 5 years**										
No	923	891	1.00	–	–	758	840	1.00	–	–
Yes	453	462	0.99	0.83	1.17	159	188	1.03	0.81	1.32
**Timing of sleep**										
Sleep before 23 h	187	182	1.00	–	–	186	149	1.00	–	–
Sleep at 23–00 h	440	429	0.99	0.77	1.28	288	328	0.70	0.53	0.94
Sleep at 00–01 h	595	593	0.96	0.75	1.23	321	436	0.61	0.46	0.81
Sleep after 01 h	48	69	0.62	0.40	0.97	37	50	0.63	0.38	1.05
**Siesta**										
Never	761	844	1.00	–	–	316	381	1.00	–	–
Ever	782	716	1.22	1.06	1.42	692	769	1.11	0.92	1.35
**Frequency of siesta (days per week)**										
Never	761	844	1.00	–	–	316	381	1.00	–	–
< 3	163	159	1.05	0.82	1.35	108	94	1.25	0.90	1.75
3–5	135	104	1.41	1.06	1.87	64	79	1.08	0.74	1.59
6–7	484	453	1.25	1.05	1.48	520	596	1.09	0.89	1.34
**Duration of siesta (min)**										
Never	761	844	1.00	–	–	316	381	1.00	–	–
< 15	115	124	1.09	0.82	1.44	82	91	1.06	0.74	1.50
15–29	163	172	1.08	0.85	1.38	141	157	1.09	0.82	1.45
30–59	233	201	1.27	1.02	1.58	191	219	1.08	0.84	1.40
60 +	271	219	1.37	1.11	1.70	278	302	1.17	0.92	1.48

Note the sum does not equal the total due to missing data.

^a^Models adjusted for age, centre, education, socioeconomic status, cigarette smoking status, family history of breast cancer in first degree relatives, BMI, physical activity, alcohol consumption, parity, age at first child, oral contraceptive, hormone replacement therapy, age at menarche, menopausal status. Categories for missing values were created for family history of breast cancer in first degree relatives, alcohol consumption, and hormone replacement therapy.

^b^Models adjusted for age, centre, education, socioeconomic status, cigarette smoking status, family history of prostate cancer in first degree relatives, BMI, physical activity, alcohol consumption.

Categories for missing values were created for family history of prostate cancer in first degree relatives and alcohol consumption.

For prostate cancer, there was also no association with categories of sleep duration (h) (Fig. 1b). There was no association among men reporting ever having a long period (at least 1 year) with sleep problems, or according to duration (years) of sleep problems, timing of sleep problems (last 5 years), or timing of sleep. Some inverse associations were observed in categories of later timing of sleep. There was no association observed among men reporting specific sleep problems, or frequent changes in the time they go to sleep (Supplemental Table 3). There was no association among men reporting ever taking siestas (OR = 1.11, 95% CI 0.92–1.35) or according to their frequency or duration (*p*ʼs for trend = > 0.05).

Results of sensitivity analysis of main findings with mutual adjustment for other sleep characteristics were virtually unchanged. Findings for ever siesta, siesta frequency or duration were unchanged with adjustment for sleep duration, sleep problems, or timing of sleep (Supplemental Table 4). Similarly, findings for sleep duration, sleep problems, and timing of sleep were unchanged with adjustment for siesta (not shown). Findings were unchanged upon exclusion of participants reporting any sleep problem in the past 5 years (not shown). There was no significant effect modification of associations of habitual sleep characteristics and either breast or prostate cancer risk by other sleep variables (sleep duration, ever sleep problems, timing of sleep, ever siesta) (results not shown, *p*ʼs > 0.05), though there was a tendency for associations with ever siesta to strengthen somewhat with increasing nighttime sleep duration for breast cancer and to a lesser extent for prostate cancer (Supplemental Table 5).

There was no evidence for effect modification of associations of sleep characteristics and either breast or prostate cancer risk according to a range of personal factors including age group, BMI, menopausal status (Supplemental Table 6), night shift work history or chronotype (Supplemental Tables 7 and 8) (*p*ʼs > 0.05). In analysis by residence outdoor blue light spectrum (participants in Barcelona and Madrid only), ORs of 1.25 (95% CI 0.80, 1.96) and 0.64 (95% CI 0.42–0.95) for breast cancer risk for timing of sleep after ≥ 00 h were observed with lower vs greater residence outdoor blue light spectrum, *p* = 0.03.

Adverse associations for ever siesta were somewhat stronger for triple-negative breast cancer (estrogen receptor (ER)-, progesterone receptor (PR)- and luminal human epidermal growth factor receptor (HER) 2-) (relative risk ratio (RRR) = 1.69, 95% CI 1.15–2.49) (*p* value heterogeneity = 0.28) (Table 3). Findings for prostate cancer were similar according to Gleason score (Table 4).

**Table 3:** Associations of selected habitual sleep characteristics and breast cancer risk by sub-phenotype, MCC-Spain, 2008–2013.

	Controls n = 1560	ER+ or PR+ and HER2− n = 1018	RRR^a^ ER+ or PR+ and HER2− vs control	LCI	UCI	HER2+ N = 270	RRR^a^ HER2+ vs control	LCI	UCI	Triple− n = 119	RRR^a^ Triple− vs control	LCI	UCI	*p* value heterogeneity
**Sleep duration (h)**														
< 6	206	132	1.08	0.82	1.43	20	0.53	0.31	0.89	6	0.41	0.17	1.02	0.01
6	304	191	1.00	0.79	1.27	52	0.89	0.61	1.30	27	1.24	0.72	2.15	0.62
7	479	309	1.00	–	–	90	1.00	–	–	32	1.00	–	–	–
8	432	299	1.06	0.86	1.32	74	0.85	0.61	1.20	42	1.43	0.88	2.34	0.23
9 +	139	87	0.98	0.71	1.35	34	1.26	0.79	2.01	12	1.14	0.56	2.36	0.67
Per 1 h			0.98	0.92	1.04		1.10	0.99	1.21		1.15	0.99	1.33	0.05
**Ever sleep problems**														
No	891	603	1.00	–	–	167	1.00	–	–	74	1.00	–	–	–
Yes	669	415	0.96	0.81	1.14	103	0.87	0.66	1.15	45	0.87	0.59	1.30	0.80
**Timing of sleep**														
Sleep before 23 h	182	130	1.00	–	–	34	1.00	–	–	11	1.00	–	–	–
Sleep at 23–00 h	429	297	0.96	0.73	1.28	67	0.90	0.57	1.43	40	1.42	0.70	2.88	0.56
Sleep at 00–01 h	593	384	0.87	0.66	1.15	116	1.16	0.75	1.79	44	1.14	0.56	2.32	0.50
Sleep after 01 h	69	33	0.60	0.36	0.98	10	0.77	0.35	1.70	2	0.42	0.09	2.01	0.76
**Siesta**														
Never	844	505	1.00	–	–	133	1.00	–	–	51	1.00	–	–	–
Ever	716	513	1.20	1.02	1.41	137	1.24	0.95	1.62	68	1.69	1.15	2.49	0.28

Note the sum does not equal the total due to missing data.

^a^Models adjusted for age, centre, education, socioeconomic status, cigarette smoking status, family history of breast cancer in first degree relatives, BMI, physical activity, alcohol consumption, parity, age at first child, oral contraceptive, hormone replacement therapy, age at menarche, menopausal status. Categories for missing values were created for family history of breast cancer in first degree relatives, alcohol consumption, and hormone replacement therapy.

**Table 4:** Associations of selected habitual sleep characteristics and prostate cancer risk by sub-phenotype, MCC-Spain, 2008–2013.

	Controls n = 1150	Gleason (6 or 7 (3 + 4)) n = 735	RRR^a^ Gleason (6 or 7 (3 + 4)) vs control	LCI	UCI	Gleason (≥ 8 or 7 (4 + 3)) n = 251	RRR^a^ Gleason (≥ 8 or 7 (4 + 3)) vs control	LCI	UCI	*p* value heterogeneity
**Sleep duration (h)**										
< 6	149	97	1.07	0.78	1.48	41	1.34	0.85	2.11	0.43
6	233	151	1.05	0.80	1.39	45	1.00	0.65	1.54	0.85
7	347	217	1.00	–	–	63	1.00	–	–	–
8	293	198	1.19	0.92	1.54	66	1.17	0.79	1.73	0.95
9 +	128	72	0.99	0.69	1.41	36	1.40	0.86	2.28	0.25
Per 1 h			1.01	0.94	1.08		1.02	0.93	1.13	0.81
**Ever sleep problems**										
No	840	554	1.00	–	–	191	1.00	–	–	–
Yes	310	181	0.90	0.72	1.13	60	0.90	0.64	1.24	0.97
**Timing of sleep**										
Sleep before 23 h	149	136	1.00	–	–	49	1.00	–	–	–
Sleep at 23–00 h	328	210	0.69	0.50	0.93	67	0.69	0.44	1.07	0.98
Sleep at 00–01 h	436	235	0.60	0.44	0.82	80	0.62	0.40	0.96	0.89
Sleep after 01 h	50	30	0.66	0.38	1.14	5	0.37	0.13	1.00	0.31
**Siesta**										
Never	381	229	1.00	–	–	79	1.00	–	–	–
Ever	769	506	1.14	0.93	1.41	172	1.07	0.79	1.45	0.72

Note the sum does not equal the total due to missing data.

^a^Models adjusted for age, centre, education, socioeconomic status, cigarette smoking status, family history of prostate cancer in first degree relatives, BMI, physical activity, alcohol consumption. Categories for missing values were created for family history of prostate cancer in first degree relatives and alcohol consumption.

Significant values are in italics.

Finally, in analysis of additional sleep characteristics captured using a subsequent supplementary circadian questionnaire in a subset of participants, there were no clear associations of any sleep characteristics around the age of 40 years including sleep duration or timing of sleep on weekdays or weekends, social jetlag, waking up at night or level of rest during sleep and either breast or prostate cancer risk (Table 5, Supplemental Table 9). However, for sleep characteristics in the previous year, there were significant positive associations for both breast and prostate cancer among participants reporting that they usually wake up at night (ORs = 1.35, 95% CI 1.10–1.65 and 1.71, 95% CI 1.28–2.27 respectively), including to urinate (ORs = 1.44, 95% CI 1.16–1.78 and 1.71, 95% CI 1.28–2.28), with somewhat stronger findings with increasing frequency per night for prostate cancer (Table 5). There was also a significant positive association among men reporting a low compared to a high level of rest during sleep in the previous year (OR = 1.55, 95% CI 1.19–2.02).

**Table 5:** Associations of sleep characteristics at either around age 40 years or in the previous year and breast and prostate cancer risk, supplemental questionnaire, MCC-Spain, 2008–2013.

	Breast cancer cases n = 1052	Breast cancer controls n = 1093	OR^a^	LCI	UCI	Prostate cancer cases n = 751	Prostate cancer controls n = 827	OR^b^	LCI	UCI
**Around age 40 years**										
Usually wake up at night										
No	827	845	1.00	–	–	590	674	1.00	–	–
Yes	225	248	0.87	0.7	1.08	161	153	1.20	0.92	1.56
If yes										
< 3 nights/wk	10	17	0.54	0.24	1.23	19	22	0.94	0.49	1.81
≥ 3 nights/wk	215	231	0.89	0.71	1.12	142	131	1.24	0.94	1.64
< 2 times/night	111	125	0.89	0.67	1.19	111	111	1.16	0.86	1.57
≥ 2 times/night	114	125	0.85	0.64	1.13	50	42	1.30	0.83	2.02
To urinate only										
No	61	79	0.76	0.53	1.10	36	33	1.23	0.73	2.06
Yes	159	166	0.92	0.71	1.18	123	117	1.20	0.90	1.61
Level of rest during sleep										
6–10 (High)	945	978	1.00	–	–	681	749	1.00	–	–
1–5 (Low)	107	115	0.89	0.66	1.18	70	78	0.90	0.63	1.28
**In the previous year**										
Usually wake up at night										
No	255	310	1.00	–	–	107	171	1.00	–	–
Yes	797	783	1.35	1.10	1.65	644	656	1.71	1.28	2.27
If yes										
< 3 nights/wk	36	29	1.25	0.73	2.14	28	38	1.19	0.67	2.10
≥ 3 nights/wk	761	754	1.35	1.10	1.66	616	618	1.75	1.31	2.33
< 2 times/night	318	313	1.35	1.06	1.72	265	308	1.43	1.05	1.94
≥ 2 times/night	479	470	1.35	1.08	1.68	379	348	2.05	1.50	2.79
To urinate only										
No	156	177	1.08	0.81	1.43	47	46	1.62	0.99	2.67
Yes	636	602	1.44	1.16	1.78	595	609	1.71	1.28	2.28
Level of rest during sleep										
6–10 (High)	769	824	1.00	–	–	576	692	1.00	–	–
1–5 (Low)	283	269	1.10	0.90	1.34	175	135	1.55	1.19	2.02

Note the sum does not equal the total due to missing data.

^a^Models adjusted for age, centre, education, socioeconomic status, cigarette smoking status, family history of breast cancer in first degree relatives, BMI, physical activity, alcohol consumption, parity, age at first child, oral contraceptive, hormone replacement therapy, age at menarche, menopausal status. Categories for missing values were created for family history of breast cancer in first degree relatives, alcohol consumption, and hormone replacement therapy.

^b^Models adjusted for age, centre, education, socioeconomic status, cigarette smoking status, family history of prostate cancer in first degree relatives, BMI, physical activity, alcohol consumption. Categories for missing values were created for family history of prostate cancer in first degree relatives and alcohol consumption.

## Discussion

Overall, there was no clear association of habitual sleep duration, sleep quality, or timing of sleep, and either breast or prostate cancer risk. There was however, a significant adverse association for breast cancer risk among women reporting ever taking siestas, which strengthened among those reporting siestas of greater frequency or duration, as well as for triple-negative disease. There was no clear association among men reporting ever taking siestas or according their frequency or duration. There were also significant positive associations in a subsequent supplementary questionnaire of usually waking up at night during the previous year with both breast and prostate cancer, as well among men reporting a low compared to a high level of rest during sleep in the previous year (but not at age 40 years).

Findings here are similar to those of previous studies reporting no clear association of either short or long sleep duration and breast or prostate cancer risk^18–20^. A meta-analysis reported a RR of 1.00 (95% CI 0.94–1.08) for breast cancer risk among those with the shortest sleep duration (ranging from < 5 to ≤ 6.5 h per night) compared with medium sleep duration, and of 1.02 (95% CI 0.92–1.12) for the longest sleep duration (ranging from > 7 to ≥ 10 h per night)^19^. Results when combining findings from previous studies of prostate cancer were 0.95 (95% CI 0.86–1.04) and 0.75 (95% CI 0.54–1.05) respectively. Results of Mendelian randomisation studies in contrast reported adverse effects of genetic variants associated with increased sleep duration and incident oestrogen receptor positive and oestrogen receptor negative breast cancer risk, suggesting that further research using objectively measured metrics of sleep duration, including biological metrics (as opposed to questionnaire data), maybe useful^40,41^.

We observed no clear associations among participants reporting having any habitual or specific types of sleep problems, with timing of sleep, or frequent changes in the timing of sleep and either breast or prostate cancer risk. Although there are fewer studies of sleep problems, the California Teachers Study reported significant trends of increasingly poor sleep quality, sleep latency, frequency of sleep disturbance, use of sleep medication, and a global sleep index and postmenopausal breast cancer risk, but not of sleep duration^22^. In the U.S. Sister Study cohort, there was no association of sleep duration and breast cancer risk, though there was a significant positive association among women reporting having difficulty sleeping ≥ 4 nights per week (HR = 1.32, 95% CI 1.09–1.61)^25^. The Trøndelag Health Study reported a significantly elevated risk of breast cancer among women reporting several insomnia symptoms simultaneously (HR = 2.38, 95% CI 1.11–5.09)^23^. Greater than 1 h of regular social jetlag was associated with a significantly increased risk of prostate cancer, particularly among men with an early chronotype, suggesting adverse effects of even mild circadian misalignment in one study^27^. In our study, there was no clear association of categories of social jetlag and either breast or prostate cancer risk (including by categories of chronotype (not shown)). Results of some studies of obstructive sleep apnea suggest elevated breast and prostate cancer risk in clinical cohorts^29,30^. Gao et al.^28^ reported associations of genetically determined obstructive sleep apnea and breast cancer risk. We had no data on sleep apnea here. Further studies with comprehensive and improved measures of sleep problems and sleep quality, captured over time are needed.

Disturbed sleep may result in melatonin suppression due to increased light at night exposure, as well as immune suppression, enhanced inflammation and cell proliferation, and alterations in oestrogen homeostatis^6,42^. There may also be indirect influences on cancer risk, with bidirectional relationships between poor sleep and increased engagement in risk behaviours^43^. Longer sleep may be related with metabolic dysfunction as well as other comorbidities^19,44^.

We observed a significant adverse association among women reporting ever taking siestas and breast cancer risk, which strengthened among those reporting siestas of greater frequency or duration, as well as for triple-negative disease (ER−, PR− and HER2−), though numbers of women here were small. Results for siesta were similar when stratified according to other sleep variables, though the greatest OR for siesta was observed among women also reporting longer habitual sleep duration. In the Million Women Study, daytime napping was also adversely associated with breast cancer risk though only in the first 4 years of follow-up, suggesting daytime napping as a marker of pre-clinical disease^45^. In the Western New York Exposures and Breast Cancer Study there was greater self-reported pre-diagnostic sleep disturbance among women with ER− and PR− disease which was greatest among women with triple negative disease, suggesting greater sleep disturbances with more aggressive disease^46^. Triple negative breast cancer is an aggressive disease with poor survival^47^. Adverse associations with siesta observed here may therefore reflect symptoms of developing/diagnosed cancer and comorbidities. More detailed data on timing of siestas and siestas over the life course was not available. A previous analysis in MCC-Spain also reported significant adverse associations of both frequent and long daytime naps with both colorectal and gastric cancer risk, particularly among those ever working night shift work^48^.

Among men, there was no clear association of ever taking siestas and prostate cancer risk. The REDUCE study reported an inverse association of daytime sleepiness and low‐grade prostate cancer risk^49^. They noted their findings may be biased by biopsy compliance. Analysis in the UK Biobank reported a lower prostate cancer risk among men reporting usually having a nap^50^. Additional studies with data on tumour characteristics, capturing detailed information on both daytime and nighttime sleep are needed. The importance of management of sleep disorders in cancer patients has been described^51^.

In this study, there were significant adverse associations with both breast and prostate cancer among participants reporting in a subsequent supplementary questionnaire that during the previous year (but not around age 40 years) they usually wake up at night, as well among men reporting a low level of rest during sleep in the previous year. Nocturia is a well established symptom of prostate cancer and related with poor sleep^20,49^. Complex bidirectional relationships of sleep and cancer have been described^51^.

There was no clear evidence for interactions of sleep characteristics with chronotype here. In the California Teachers Study, some evidence of interaction of sleep deficiency and chronotype was observed^22^. A recent analysis in the EPICAP study reported adverse associations of sleep deprivation and prostate cancer risk among men with an evening chronotype^52^. Further research including information on chronotype and other circadian-related factors is needed.

Strengths of this study include its large-scale population-based design with detailed data on a range of habitual daytime and nighttime sleep characteristics including sleep duration, sleep quality, timing of sleep, and siesta, addressing limited data on sleep characteristics in previous studies which have focussed mainly on sleep duration^31,32^. Detailed data on potential confounding factors, disease subtypes, as well as on other interrelated factors including night shift work, chronotype, and outdoor light at night were also captured here.

Limitations include the self-reported retrospective nature of data captured on habitual sleep characteristics. Although there was limited information available to examine reporting of retrospective sleep habits due to differences in questions and questionnaires used to capture sleep data for different points in the lifetime, comparison of agreement of habitual hours of sleep duration (< 7, 7, ≥ 8 h) reported in the main study questionnaire at recruitment to that in the subsequent supplemental circadian questionnaire for age 40 years (weekdays) was generally poor (% agreement 41–45%, Cohenʼs kappa 0.11–0.14) (note sleep duration was assessed differently in the two questionnaires (see above)). Agreement for timing of sleep (before 00 h vs after 00 h) was fair (% agreement 45–57%, Cohenʼs kappa 0.20–0.32). Further studies with prospectively measured data are needed^51^. Other metrics of sleep have also been suggested including indices of lifetime sleep or objectively measured sleep fragmentation for example.

It is also unclear to what extent potential selection biases may have affected findings here, participation rates were moderate for the main interview, and were reduced further for completion of the later subsequent supplemental circadian questionnaire. There was a tendency for participants with a lower level of education to be less likely to complete the supplemental circadian questionnaire (19.11% of female and 29.8% of male participants had a less than primary school education among those who did not complete the supplemental circadian questionnaire vs 15.1% and 20.4% of total participants). There were also multiple comparisons performed and some findings maybe due to chance.

## Conclusions

There was no clear association of various personal habitual sleep characteristics and either breast or prostate cancer risk. Although there were some adverse associations observed with some sleep characteristics including siesta, or waking up at night and a low level of rest during sleep in the previous year, they likely reflect symptoms of developing/diagnosed disease. Further research with prospective and validated markers of sleep is needed.

## Supplementary Information

Supplementary Tables.

## Data Availability

The datasets used and/or analysed during the current study are available from the corresponding author on reasonable request.

## Abbreviations

BMI: Body mass index
CI: Confidence interval
ER: Estrogen receptor
ICD-10: International Classification of Diseases 10
HER: Luminal human epidermal growth factor receptor
MET: Metabolic equivalent
PR: Progesterone receptor
OR: Odds ratio
RRR: Relative risk ratio

## References

[1] Chattu VK, Sakhamuri SM, Kumar R, Spence DW, BaHammam AS, Pandi-Perumal SR (2018). Insufficient sleep syndrome: Is it time to classify it as a major noncommunicable disease?. Sleep Sci..

[2] Kocevska D, Lysen TS, Dotinga A, Koopman-Verhoeff ME, Luijk MPCM, Antypa N, Biermasz NR, Blokstra A, Brug J, Burk WJ, Comijs HC, Corpeleijn E, Dashti HS, de Bruin EJ, de Graaf R, Derks IPM, Dewald-Kaufmann JF, Elders PJM, Gemke RJBJ, Grievink L, Hale L, Hartman CA, Heijnen CJ, Huisman M, Huss A, Ikram MA, Jones SE, Velderman MK, Koning M, Meijer AM, Meijer K, Noordam R, Oldehinkel AJ, Groeniger JO, Penninx BWJH, Picavet HSJ, Pieters S, Reijneveld SA, Reitz E, Renders CM, Rodenburg G, Rutters F, Smith MC, Singh AS, Snijder MB, Stronks K, Ten Have M, Twisk JWR, Van de Mheen D, van der Ende J, van der Heijden KB, van der Velden PG, van Lenthe FJ, van Litsenburg RRL, van Oostrom SH, van Schalkwijk FJ, Sheehan CM, Verheij RA, Verhulst FC, Vermeulen MCM, Vermeulen RCH, Verschuren WMM, Vrijkotte TGM, Wijga AH, Willemen AM, Ter Wolbeek M, Wood AR, Xerxa Y, Bramer WM, Franco OH, Luik AI, Van Someren EJW, Tiemeier H (2021). Sleep characteristics across the lifespan in 1.1 million people from the Netherlands, United Kingdom and United States: A systematic review and meta-analysis. Nat. Hum. Behav..

[3] Shen X, Wu Y, Zhang D (2016). Nighttime sleep duration, 24-hour sleep duration and risk of all-cause mortality among adults: A meta-analysis of prospective cohort studies. Sci. Rep..

[4] Yin J, Jin X, Shan Z, Li S, Huang H, Li P, Peng X, Peng Z, Yu K, Bao W, Yang W, Chen X, Liu L (2017). Relationship of sleep duration with all-cause mortality and cardiovascular events: A systematic review and dose-response meta-analysis of prospective cohort studies. J. Am. Heart Assoc..

[5] Ferlay J, Colombet M, Soerjomataram I, Parkin DM, Piñeros M, Znaor A, Bray F (2021). Cancer statistics for the year 2020: An overview. Int. J. Cancer..

[6] IARC Monographs Vol 124 Group (2019). Carcinogenicity of night shift work. Lancet Oncol..

[7] Cao J, Eshak ES, Liu K, Muraki I, Cui R, Iso H, Tamakoshi A, JACC Study Group (2019). Sleep duration and risk of breast cancer: The JACC Study. Breast Cancer Res. Treat..

[8] Gapstur SM, Diver WR, Stevens VL, Carter BD, Teras LR, Jacobs EJ (2014). Work schedule, sleep duration, insomnia, and risk of fatal prostate cancer. Am. J. Prev. Med..

[9] Kakizaki M, Inoue K, Kuriyama S, Sone T, Matsuda-Ohmori K, Nakaya N, Fukudo S, Tsuji I (2008). Sleep duration and the risk of prostate cancer: The Ohsaki Cohort Study. Br. J. Cancer.

[10] Wang P, Ren FM, Lin Y, Su FX, Jia WH, Su XF, Tang LY, Ren ZF (2015). Night-shift work, sleep duration, daytime napping, and breast cancer risk. Sleep Med..

[11] Xiao Q, Signorello LB, Brinton LA, Cohen SS, Blot WJ, Matthews CE (2016). Sleep duration and breast cancer risk among black and white women. Sleep Med..

[12] Hurley S, Goldberg D, Bernstein L, Reynolds P (2015). Sleep duration and cancer risk in women. Cancer Causes Control.

[13] Pinheiro SP, Schernhammer ES, Tworoger SS, Michels KB (2006). A prospective study on habitual duration of sleep and incidence of breast cancer in a large cohort of women. Cancer Res..

[14] Lozano-Lorca M, Olmedo-Requena R, Vega-Galindo MV, Vázquez-Alonso F, Jiménez-Pacheco A, Salcedo-Bellido I, Sánchez MJ, Jiménez-Moleón JJ (2020). Night shift work, chronotype, sleep duration, and prostate cancer risk: CAPLIFE study. Int. J. Environ. Res. Public Health.

[15] McNeil J, Heer E, Willemsen RF, Friedenreich CM, Brenner DR (2020). The effects of shift work and sleep duration on cancer incidence in Alberta’s Tomorrow Project cohort. Cancer Epidemiol..

[16] Shen J, Chrisman M, Wu X, Chow WH, Zhao H (2019). Sleep duration and risk of cancer in the Mexican American Mano-a-Mano Cohort. Sleep Health.

[17] Shigesato M, Kawai Y, Guillermo C, Youkhana F, Shvetsov YB, Setiawan VW, Haiman CA, Le Marchand L, Maskarinec G (2020). Association between sleep duration and breast cancer incidence: The multiethnic cohort. Int. J. Cancer.

[18] Wong ATY, Heath AK, Tong TYN, Reeves GK, Floud S, Beral V, Travis RC (2021). Sleep duration and breast cancer incidence: Results from the Million Women Study and meta-analysis of published prospective studies. Sleep.

[19] Chen Y, Tan F, Wei L, Li X, Lyu Z, Feng X, Wen Y, Guo L, He J, Dai M, Li N (2018). Sleep duration and the risk of cancer: A systematic review and meta-analysis including dose-response relationship. BMC Cancer.

[20] Liu R, Wu S, Zhang B, Guo M, Zhang Y (2020). The association between sleep duration and prostate cancer: A systematic review and meta-analysis. Medicine (Baltimore).

[21] Chung WS, Lin CL (2019). Sleep disorders associated with risk of prostate cancer: A population-based cohort study. BMC Cancer.

[22] Hurley S, Goldberg D, Von Behren J, Clague DeHart J, Wang S, Reynolds P (2020). Sleep deficiency and breast cancer risk among postmenopausal women in the California teachers study (CTS). Cancer Causes Control.

[23] Sen A, Opdahl S, Strand LB, Vatten LJ, Laugsand LE, Janszky I (2017). Insomnia and the risk of breast cancer: The HUNT Study. Psychosom. Med..

[24] Sigurdardottir LG, Valdimarsdottir UA, Mucci LA, Fall K, Rider JR, Schernhammer E, Czeisler CA, Launer L, Harris T, Stampfer MJ, Gudnason V, Lockley SW (2013). Sleep disruption among older men and risk of prostate cancer. Cancer Epidemiol. Biomark. Prev..

[25] White AJ, Weinberg CR, Park YM, D'Aloisio AA, Vogtmann E, Nichols HB, Sandler DP (2017). Sleep characteristics, light at night and breast cancer risk in a prospective cohort. Int. J. Cancer.

[26] Yang W, Shi Y, Ke X, Sun H, Guo J, Wang X (2019). Long-term sleep habits and the risk of breast cancer among Chinese women: A case–control study. Eur. J. Cancer Prev..

[27] Hu L, Harper A, Heer E, McNeil J, Cao C, Park Y, Martell K, Gotto G, Shen-Tu G, Peters C, Brenner D, Yang L (2021). Social jetlag and prostate cancer incidence in Alberta's Tomorrow Project: A prospective cohort study. Cancers (Basel).

[28] Gao XL, Jia ZM, Zhao FF, An DD, Wang B, Cheng EJ, Chen Y, Gong JN, Liu D, Huang YQ, Yang JJ, Wang SJ (2020). Obstructive sleep apnea syndrome and causal relationship with female breast cancer: A mendelian randomization study. Aging (Albany NY).

[29] Jara SM, Phipps AI, Maynard C, Weaver EM (2020). The association of sleep apnea and cancer in veterans. Otolaryngol. Head Neck Surg..

[30] Kendzerska T, Povitz M, Leung RS, Boulos MI, McIsaac DI, Murray BJ, Bryson GL, Talarico R, Hilton JF, Malhotra A, Gershon AS (2021). Obstructive sleep apnea and incident cancer: A large retrospective multicenter clinical cohort study. Cancer Epidemiol. Biomark. Prev..

[31] Samuelsson LB, Bovbjerg DH, Roecklein KA, Hall MH (2018). Sleep and circadian disruption and incident breast cancer risk: An evidence-based and theoretical review. Neurosci. Biobehav. Rev..

[32] Wendeu-Foyet MG, Menegaux F (2017). Circadian disruption and prostate cancer risk: An updated review of epidemiological evidences. Cancer Epidemiol. Biomark. Prev..

[33] Castaño-Vinyals G, Aragonés N, Pérez-Gómez B, Martíne V, Llorca J, Moreno V, Altzibar JM, Ardanaz E, de Sanjosé S, Jiménez-Moleón JJ, Tardón A, Alguacil J, Peiró R, Marcos-Gragera R, Navarro C, Pollán M, Kogevinas M, MCC-Spain Study Group (2015). Population-based multicase-control study in common tumors in Spain (MCC-Spain): Rationale and study design. Gac Sanit..

[34] WHO (1992). International Statistical Classification of Diseases and Related Health Problems, 10th Revision.

[35] Roenneberg T, Wirz-Justice A, Merrow M (2003). Life between clocks: Daily temporal patterns of human chronotypes. J. Biol. Rhythms.

[36] Papantoniou K, Castaño-Vinyals G, Espinosa A, Aragonés N, Pérez-Gómez B, Burgos J, Gómez-Acebo I, Llorca J, Peiró R, Jimenez-Moleon JJ, Arredondo F, Tardon A, Pollan M, Kogevinas M (2015). Night shift work, chronotpe and prostate cancer risk in the MCC-Spain case–control study. Int. J. Cancer.

[37] Papantoniou K, Castaño-Vinyals G, Espinosa A, Aragonés N, Pérez-Gómez B, Ardanaz E, Altzibar JM, Sanchez VM, Gómez-Acebo I, Llorca J, Muñoz D, Tardón A, Peiró R, Marcos-Gragera R, Pollan M, Kogevinas M (2016). Breast cancer risk and night shift work in a case–control study in a Spanish population. Eur. J. Epidemiol..

[38] Garcia-Saenz A, Sánchez de Miguel A, Espinosa A, Valentin A, Aragonés N, Llorca J, Amiano P, Martín Sánchez V, Guevara M, Capelo R, Tardón A, Peiró-Perez R, Jiménez-Moleón JJ, Roca-Barceló A, Pérez-Gómez B, Dierssen-Sotos T, Fernández-Villa T, Moreno-Iribas C, Moreno V, García-Pérez J, Castaño-Vinyals G, Pollán M, Aubé M, Kogevinas M (2018). Evaluating the association between artificial light-at-night exposure and breast and prostate cancer risk in Spain (MCC-Spain Study). Environ. Health Perspect..

[39] R Core Team. *R: A Language and Environment for Statistical Computing*. https://www.R-project.org/ (R Foundation for Statistical Computing, 2018).

[40] Richmond RC, Anderson EL, Dashti HS, Jones SE, Lane JM, Strand LB, Brumpton B, Rutter MK, Wood AR, Straif K, Relton CL, Munafò M, Frayling TM, Martin RM, Saxena R, Weedon MN, Lawlor DA, Smith GD (2019). Investigating causal relations between sleep traits and risk of breast cancer in women: Mendelian randomisation study. BMJ.

[41] Chen F, Wen W, Long J, Shu X, Yang Y, Shu XO, Zheng W (2022). Mendelian randomization analyses of 23 known and suspected risk factors and biomarkers for breast cancer overall and by molecular subtypes. Int. J. Cancer.

[42] Gozal D, Farré R, Nieto FJ (2016). Obstructive sleep apnea and cancer: Epidemiologic links and theoretical biological constructs. Sleep Med. Rev..

[43] Taber JM, Cribbet MR, Cadmus-Bertram L, Mays D, Smith MEB, Rana B, Paljarvi T (2021). Associations among sleep and cancer risk behaviors: A scoping review of experimental studies in healthy adult populations. Int. J. Behav. Med..

[44] Beaman A, Bhide MC, McHill AW, Thosar SS (2021). Biological pathways underlying the association between habitual long-sleep and elevated cardiovascular risk in adults. Sleep Med..

[45] Cairns BJ, Travis RC, Wang XS, Reeves GK, Green J, Beral V (2012). A short-term increase in cancer risk associated with daytime napping is likely to reflect pre-clinical disease: Prospective cohort study. Br. J. Cancer.

[46] Vaughn CB, Freudenheim JL, Nie J, Sucheston-Campbell L, Wactawski-Wende J, Marian C, Shields PG, Kallakury BV, Trevisan M, Ochs-Balcom HM (2018). Sleep and breast cancer in the Western New York exposures and breast cancer (WEB) study. J. Clin. Sleep Med..

[47] Howard FM, Olopade OE (2021). Epidemiology of triple-negative breast cancer: A review. Cancer J..

[48] Papantoniou K, Castaño-Vinyals G, Espinosa A, Turner MC, Martín-Sánchez V, Casabonne D, Aragonés N, Gómez-Acebo I, Ardanaz E, Jimenez-Moleon JJ, Amiano P, Molina-Barceló A, Alguacil J, Fernández-Tardón G, Huerta JM, Hernández-Segura N, Perez-Gomez B, Llorca J, Vidán-Alli J, Olmedo-Requena R, Gil L, Castañon-López C, Pollan M, Kogevinas M, Moreno V (2021). Sleep duration and napping in relation to colorectal and gastric cancer in the MCC-Spain study. Sci. Rep..

[49] Wiggins EK, Oyekunle T, Howard LE, Markt SC, Mucci LA, Bliwise DL, Moreira DM, Andriole GL, Hopp ML, Freedland SJ, Allott EH (2020). Sleep quality and prostate cancer aggressiveness: Results from the REDUCE trial. Prostate.

[50] Lv X, Li Y, Li R, Guan X, Li L, Li J, Si S, Ji X, Cao Y, Xue F (2022). Relationships of sleep traits with prostate cancer risk: A prospective study of 213,999 UK Biobank participants. Prostate.

[51] Mogavero MP, DelRosso LM, Fanfulla F, Bruni O, Ferri R (2021). Sleep disorders and cancer: State of the art and future perspectives. Sleep Med. Rev..

[52] Cordina-Duverger E, Cénée S, Trétarre B, Rebillard X, Lamy PJ, Wendeu-Foyet G, Menegaux F (2022). Sleep patterns and risk of prostate cancer: A population-based case control study in France (EPICAP). Cancer Epidemiol. Biomark. Prev..

