# Supplementary material for: Sleep and breast and prostate cancer risk in the MCC-Spain study
Source: Sci Rep. 2022 Dec 16;12:21807. doi: 10.1038/s41598-022-25789-9 (PMC9758125; doi:10.1038/s41598-022-25789-9)
Supplement: Supplementary file 1 — Supplementary Tables. [file 41598_2022_25789_MOESM1_ESM.docx]

**Sleep and Breast and Prostate Cancer Risk in the MCC-Spain Study**

Michelle C Turner^1-3^, Esther Gracia-Lavedan^1-3^, Kyriaki Papantoniou^4^, Nuria Aragonés^3,5^, Gemma Castaño-Vinyals^1-3,6^, Trinidad Dierssen-Sotos^3,7,8^, Pilar Amiano^3,9,10^, Eva Ardanaz^3,11^, Alba Marcos Delgado^12^, Ana Molina Barceló^13^, Juan Alguacil^3,14^, Yolanda Benavente^3,15^, Thalia Belmonte^16^, José J Jiménez-Moleón^3,17,18^, Rafael Marcos-Gragera^3,19-22^, Beatriz Pérez^23^, Inés Gómez-Acebo^3,7,8^, Marina Pollán^3,23^, Manolis Kogevinas^1-3,6^

Supplemental Table 1. Distribution of participant characteristics, breast cancer controls (n = 1,609), by categories of habitual sleep duration (hours), and siesta, MCC-Spain, 2008–2013.

|  | <6h  (n=213)  % | 6h  (n=313)  % | 7h  (n=490)  % | 8h  (n=445)  % | 9h+  (n=148)  % | *p* value | Never Siesta (n=883)  % | Ever  Siesta  (n=726)  % | *p* value |
| --- | --- | --- | --- | --- | --- | --- | --- | --- | --- |
| Age (Years), Mean (SD) | 62.7 (12.4) | 58.3 (12.7) | 55.7 (12.5) | 57.2 (14.1) | 64.0 (12.8) | *<0.001* | 57.6 (13.2) | 59.2 (13.4) | *0.01* |
| Centre |  |  |  |  |  | *<0.001* |  |  | *0.001* |
| Asturias | 7.5% | 9.6% | 5.9% | 7.2% | 4.7% |  | 7.6% | 6.5% |  |
| Barcelona | 16.9% | 16.3% | 12.2% | 12.4% | 20.3% |  | 15.1% | 13.6% |  |
| Cantabria | 6.6% | 12.1% | 10.8% | 15.5% | 4.1% |  | 12.1% | 10.1% |  |
| Girona | 1.4% | 1.6% | 5.1% | 3.6% | 4.1% |  | 3.9% | 2.9% |  |
| Granada | - | - | - | - | - |  | *-* | *-* |  |
| Guipuzcoa | 9.9% | 10.5% | 15.1% | 14.2% | 11.5% |  | 10.8% | 15.6% |  |
| Huelva | 3.3% | 3.8% | 4.3% | 4.3% | 5.4% |  | 3.4% | 5.1% |  |
| Leon | 15.5% | 8.0% | 9.4% | 12.6% | 18.2% |  | 13.0% | 9.9% |  |
| Madrid | 24.4% | 24.9% | 22.7% | 15.5% | 18.9% |  | 22.4% | 19.3% |  |
| Navarra | 10.8% | 9.9% | 9.8% | 10.6% | 9.5% |  | 8.4% | 12.3% |  |
| Valencia | 3.8% | 3.2% | 4.7% | 4.3% | 3.4% |  | 3.40% | 4.8% |  |
| Education |  |  |  |  |  | *<0.001* |  |  | *0.85* |
| < Primary | 27.7% | 13.7% | 9.0% | 14.8% | 37.2% |  | 17.2% | 15.8% |  |
| Primary | 37.6% | 39.6% | 25.7% | 30.1% | 29.7% |  | 31.8% | 31.3% |  |
| Secondary | 23.0% | 26.8% | 36.7% | 31.9% | 21.6% |  | 29.7% | 31.0% |  |
| University | 11.7% | 19.8% | 28.6% | 23.1% | 11.5% |  | 21.3% | 21.9% |  |
| Socioeconomic Score |  |  |  |  |  | *<0.001* |  |  | *0.84* |
| Low (0-2) | 45.1% | 33.5% | 22.4% | 29.9% | 47.3% |  | 30.9% | 33.2% |  |
| Medium (3-5) | 46.5% | 48.9% | 53.5% | 48.5% | 39.9% |  | 48.9% | 49.2% |  |
| High (6-7) | 6.6% | 15.7% | 23.3% | 20.2% | 8.1% |  | 17.2% | 17.5% |  |
| Cigarette Smoking |  |  |  |  |  | *<0.001* |  |  | *0.31* |
| Never | 67.1% | 56.5% | 52.0% | 59.8% | 75.7% |  | 60.4% | 57.9% |  |
| Former | 15.0% | 18.2% | 23.7% | 18.2% | 17.6% |  | 19.6% | 19.1% |  |
| Current | 17.8% | 24.9% | 24.3% | 21.8% | 6.8% |  | 19.8% | 23.0% |  |
| Family History of Breast Cancer |  |  |  |  |  | *0.68* |  |  | *0.98* |
| None | 88.7% | 86.9% | 88.0% | 88.3% | 85.8% |  | 87.8% | 87.7% |  |
| Any | 8.0% | 8.3% | 9.8% | 7.2% | 10.1% |  | 8.49% | 8.68% |  |
| BMI (kg/m^2^) |  |  |  |  |  | *<0.001* |  |  | *0.78* |
| <25 | 41.3% | 44.4% | 59.0% | 51.2% | 42.6% |  | 50.4% | 49.9% |  |
| 25-<30 | 36.2% | 33.5% | 28.6% | 31.7% | 31.8% |  | 31.0% | 32.5% |  |
| >=30 | 22.5% | 22.0% | 12.4% | 17.1% | 25.7% |  | 18.6% | 17.6% |  |
| Physical activity |  |  |  |  |  | *0.10* |  |  | *0.59* |
| Inactive | 46.5% | 45.0% | 35.7% | 37.8% | 47.3% |  | 41.7% | 39.3% |  |
| Slightly active | 15.5% | 15.3% | 21.4% | 19.3% | 14.9% |  | 18.7% | 17.8% |  |
| Moderately active | 10.8% | 11.2% | 12.9% | 12.6% | 14.2% |  | 11.7% | 13.1% |  |
| Very Active | 27.2% | 28.4% | 30.0% | 30.3% | 23.6% |  | 28.0% | 29.9% |  |
| Alcohol consumption^a^ |  |  |  |  |  | *<0.001* |  |  | *<0.001* |
| <median | 50.2% | 44.7% | 38.6% | 43.4% | 54.1% |  | 47.7% | 39.7% |  |
| ≥median | 34.7% | 43.5% | 50.4% | 44.3% | 32.4% |  | 39.0% | 49.3% |  |
| Parity |  |  |  |  |  | *<0.001* |  |  | *0.15* |
| Nulliparous | 11.7% | 19.2% | 20.6% | 23.1% | 14.2% |  | 17.7% | 21.2% |  |
| 1-2 chilldren | 50.7% | 57.8% | 58.0% | 55.1% | 45.9% |  | 56.9% | 52.9% |  |
| 3+ children | 36.6% | 23.0% | 21.2% | 21.1% | 39.9% |  | 25.0% | 25.6% |  |
| Age at First Child |  |  |  |  |  | *<0.001* |  |  | *0.01* |
| Nulliparous | 11.7% | 19.2% | 20.6% | 23.1% | 14.2% |  | 17.7% | 21.2% |  |
| <20 years | 3.3% | 4.5% | 3.7% | 3.6% | 6.76% |  | 5.6% | 2.2% |  |
| 20-24 years | 36.6% | 27.2% | 19.2% | 20.4% | 28.4% |  | 24.2% | 24.2% |  |
| 25-29 years | 30.5% | 30.7% | 32.4% | 32.1% | 35.1% |  | 32.3% | 31.7% |  |
| >29 years | 16.9% | 18.2% | 23.7% | 20.0% | 14.9% |  | 19.7% | 20.1% |  |
| Age at menarche |  |  |  |  |  | *0.33* |  |  | *0.88* |
| <12 years | 20.7% | 20.4% | 19.8% | 16.9% | 14.2% |  | 18.9% | 18.5% |  |
| 12-13 years | 21.1% | 21.7% | 25.9% | 24.3% | 18.9% |  | 22.9% | 24.0% |  |
| 14+ years | 55.4% | 55.6% | 52.4% | 56.6% | 61.5% |  | 54.5% | 56.6% |  |
| Oral Contraceptive Use |  |  |  |  |  | *<0.001* |  |  | *0.42* |
| Never | 57.3% | 48.6% | 45.3% | 53.9% | 63.5% |  | 50.6% | 52.8% |  |
| Ever | 42.3% | 51.4% | 54.7% | 45.8% | 36.5% |  | 49.3% | 47.1% |  |
| Hormone Replacement Therapy |  |  |  |  |  | *0.32* |  |  | *0.33* |
| Never | 85.9% | 89.8% | 89.0% | 91.7% | 90.5% |  | 90.3% | 88.8% |  |
| Ever | 7.5% | 5.1% | 8.8% | 6.5% | 5.4% |  | 6.3% | 7.7% |  |
| Menopausal Status |  |  |  |  |  | *<0.001* |  |  | *0.12* |
| Premenopausal | 85.0% | 70.0% | 59.4% | 67.6% | 79.7% |  | 67.3% | 71.1% |  |
| Postmenopausal | 15.0% | 30.0% | 40.4% | 32.4% | 20.3% |  | 32.6% | 28.9% |  |
| Night Shift Work |  |  |  |  |  | *<0.001* |  |  | *0.23* |
| Never | 67.1% | 69.3% | 77.8% | 70.3% | 60.1% |  | 71.7% | 70.2% |  |
| Ever | 11.7% | 12.8% | 11.6% | 10.6% | 6.8% |  | 10.0% | 12.5% |  |
| Housewife | 15.5% | 13.4% | 7.8% | 15.3% | 22.3% |  | 12.6% | 14.2% |  |
| Chronotype |  |  |  |  |  | *0.62* |  |  | *0.88* |
| Morning | 35.7% | 33.5% | 34.1% | 29.9% | 28.4% |  | 32.4% | 32.6% |  |
| Neither | 29.1% | 35.1% | 32.2% | 33.7% | 23.6% |  | 32.0% | 32.0% |  |
| Evening | 16.9% | 15.3% | 18.6% | 19.1% | 16.9% |  | 18.2% | 17.1% |  |
| Outdoor blue light spectrum^b^ |  |  |  |  |  | *0.37* |  |  | *0.68* |
| < median (0.15) | 51.3% | 53.7% | 50.6% | 50.0% | 36.2% |  | 49.1% | 51.5% |  |
| ≥ median (0.15) | 48.7% | 46.3% | 49.4% | 49.0% | 63.8% |  | 50.9% | 48.5% |  |

Note in some cases the sum does not equal the total due to missing data.

^a^ Median = 1.63 ethanol g/day in women.

^b^ Participants in Barcelona and Madrid only, n=491 included breast cancer controls, current address

Supplemental Table 2. Distribution of participant characteristics, prostate cancer controls (n = 1,179), by categories of habitual sleep duration (hours), and ever siesta, MCC-Spain, 2008–2013.

|  | <6h  (n=152)  % | 6h  (n=242)  % | 7h  (n=352)  % | 8h  (n=302)  % | 9h+  (n=131)  % | *p* value | Never Siesta  (n=405)  % | Ever  Siesta  (n=774)  % | *p* value |
| --- | --- | --- | --- | --- | --- | --- | --- | --- | --- |
| Age (Years), Mean (SD) | 66.1 (7.85) | 65.4 (8.40) | 64.4 (8.89) | 67.7 (7.33) | 71.1 (6.79) | *<0.001* | 66.6 (9.01) | 66.3 (7.92) | *0.53* |
| Centre |  |  |  |  |  | *<0.001* |  |  | *<0.001* |
| Asturias | 6.6% | 3.7% | 5.4% | 8.3% | 17.6% |  | 10.9% | 5.4% |  |
| Barcelona | 30.3% | 37.2% | 26.7% | 30.5% | 25.2% |  | 29.6% | 30.4% |  |
| Cantabria | 4.6% | 11.6% | 17.6% | 18.9% | 10.7% |  | 17.8% | 12.4% |  |
| Girona | - | - | - | - | - |  | - | - |  |
| Granada | 14.5% | 7.4% | 10.5% | 7.3% | 10.7% |  | 7.7% | 10.6% |  |
| Guipuzcoa | - | - | - | - | - |  | - | - |  |
| Huelva | 11.2% | 6.2% | 5.4% | 6.6% | 8.4% |  | 3.0% | 9.0% |  |
| Leon | - | - | - | - | - |  | - | - |  |
| Madrid | 25.0% | 27.3% | 28.4% | 22.8% | 22.1% |  | 26.9% | 24.9% |  |
| Navarra | - | - | - | - | - |  | - | - |  |
| Valencia | 7.9% | 6.6% | 6.0% | 5.6% | 5.3% |  | 4.2% | 7.2% |  |
| Education |  |  |  |  |  | *<0.001* |  |  | *0.11* |
| < Primary | 23.0% | 16.5% | 14.2% | 18.2% | 32.8% |  | 22.7% | 16.9% |  |
| Primary | 37.5% | 31.8% | 34.9% | 32.8% | 42.0% |  | 33.1% | 35.8% |  |
| Secondary | 27.0% | 27.3% | 30.1% | 28.8% | 19.1% |  | 26.9% | 27.9% |  |
| University | 12.5% | 24.4% | 20.7% | 20.2% | 6.1% |  | 17.3% | 19.4% |  |
| Socioeconomic Score |  |  |  |  |  | *<0.001* |  |  | *0.31* |
| Low (0-2) | 38.8% | 32.6% | 36.6% | 31.5% | 53.4% |  | 38.3% | 35.8% |  |
| Medium (3-5) | 50.0% | 46.3% | 49.1% | 48.7% | 38.9% |  | 43.2% | 49.6% |  |
| High (6-7) | 9.21% | 17.4% | 13.4% | 17.2% | 5.3% |  | 13.1% | 14.1% |  |
| Cigarette Smoking |  |  |  |  |  | *0.04* |  |  | *0.04* |
| Never | 29.6% | 18.6% | 31.5% | 27.5% | 30.5% |  | 31.9% | 25.2% |  |
| Former | 44.7% | 55.4% | 48.6% | 48.0% | 49.6% |  | 47.4% | 50.5% |  |
| Current | 25.7% | 26.0% | 19.3% | 24.2% | 19.8% |  | 20.2% | 24.2% |  |
| Family History of Prostate Cancer |  |  |  |  |  | *0.97* |  |  | *0.66* |
| None | 90.8% | 85.5% | 88.9% | 87.4% | 90.1% |  | 87.9% | 88.4% |  |
| Any | 5.3% | 6.6% | 6.3% | 5.6% | 6.9% |  | 6.7% | 5.8% |  |
| BMI (kg/m^2^) |  |  |  |  |  | *0.14* |  |  | *0.08* |
| <25 | 28.3% | 20.7% | 23.3% | 28.5% | 21.4% |  | 28.4% | 22.5% |  |
| 25-<30 | 47.4% | 53.3% | 55.4% | 48.0% | 47.3% |  | 49.1% | 52.2% |  |
| >=30 | 24.3% | 26.0% | 21.3% | 23.5% | 31.3% |  | 22.5% | 25.3% |  |
| Physical activity |  |  |  |  |  | *0.30* |  |  | *0.21* |
| Inactive | 43.4% | 39.7% | 38.6% | 42.4% | 42.7% |  | 42.5% | 40.1% |  |
| Slightly active | 13.8% | 14.5% | 9.4% | 14.6% | 9.2% |  | 9.6% | 13.7% |  |
| Moderately active | 9.2% | 12.8% | 13.9% | 8.3% | 11.5% |  | 10.9% | 11.6% |  |
| Very Active | 33.6% | 33.1% | 38.1% | 34.8% | 36.6% |  | 37.0% | 34.6% |  |
| Alcohol consumption^a^ |  |  |  |  |  | *0.34* |  |  | *0.09* |
| <median | 46.7% | 46.3% | 42.3% | 41.1% | 43.5% |  | 47.4% | 41.5% |  |
| ≥median | 34.9% | 41.7% | 46.9% | 45.0% | 43.5% |  | 40.7% | 44.8% |  |
| Night Shift Work |  |  |  |  |  | *0.003* |  |  | *0.25* |
| Never | 77.6% | 71.9% | 75.3% | 72.2% | 58.8% |  | 67.4% | 74.8% |  |
| Ever | 20.4% | 24.4% | 23.3% | 24.8% | 38.9% |  | 26.4% | 24.7% |  |
| Chronotype |  |  |  |  |  | *0.16* |  |  | *0.23* |
| Morning | 43.4% | 43.0% | 41.5% | 37.7% | 29.0% |  | 40.7% | 39.1% |  |
| Neither | 19.7% | 24.8% | 30.1% | 31.8% | 30.5% |  | 24.2% | 30.2% |  |
| Evening | 7.9% | 9.5% | 12.2% | 9.9% | 9.9% |  | 9.6% | 10.6% |  |
| Outdoor blue light spectrum^b^ |  |  |  |  |  | *0.14* |  |  | *1.00* |
| < median (0.15) | 62.3% | 50.3% | 45.9% | 47.1% | 54.4% |  | 50.2% | 50.0% |  |
| ≥ median (0.15) | 37.7% | 49.7% | 54.1% | 52.9% | 45.6% |  | 49.8% | 50.0% |  |

Note in some cases the sum does not equal the total due to missing values.

^a^ Median = 18.8 ethanol g/day in men.

^b^ Participants in Barcelona and Madrid only, n=623 included prostate cancer controls, current address

Supplemental Table 3. Associations of specific types of habitual sleep problems and breast and prostate cancer risk, MCC-Spain, 2008–2013.

|  | Breast Cancer Cases  n=1,543 | Breast Cancer Controls  n=1,560 | OR^a^ | LCI | UCI | Prostate Cancer Cases  n=1,008 | Prostate Cancer Controls  n=1,150 | OR^b^ | LCI | UCI |
| --- | --- | --- | --- | --- | --- | --- | --- | --- | --- | --- |
| Problems Falling Asleep |  |  |  |  |  |  |  |  |  |  |
| No | 923 | 891 | 1.00 | - | - | 758 | 840 | 1.00 | - | - |
| Yes | 447 | 481 | 0.96 | 0.81 | 1.13 | 153 | 177 | 1.00 | 0.78 | 1.28 |
| Waking Up in the Middle of the Night |  |  |  |  |  |  |  |  |  |  |
| No | 923 | 891 | 1.00 | - | - | 758 | 840 | 1.00 | - | - |
| Yes | 485 | 522 | 0.96 | 0.82 | 1.13 | 194 | 225 | 0.99 | 0.79 | 1.24 |
| Taking Medication to Fall Asleep |  |  |  |  |  |  |  |  |  |  |
| No | 923 | 891 | 1.00 | - | - | 758 | 840 | 1.00 | - | - |
| Yes | 411 | 414 | 1.07 | 0.89 | 1.27 | 123 | 151 | 0.94 | 0.71 | 1.23 |
| Frequent Changes in Time of Sleep |  |  |  |  |  |  |  |  |  |  |
| No | 1,344 | 1,334 | 1.00 | - | - | 733 | 863 | 1.00 | - | - |
| Yes | 199 | 226 | 0.91 | 0.73 | 1.13 | 275 | 287 | 1.13 | 0.92 | 1.39 |

Note the sum does not equal the total due to missing data.

^a^ Models adjusted for age, centre, education, socioeconomic status, cigarette smoking status, family history of breast cancer in first degree relatives, BMI, physical activity, alcohol consumption, parity, age at first child, oral contraceptive, hormone replacement therapy, age at menarche, menopausal status. Categories for missing values were created for family history of breast cancer in first degree relatives, alcohol consumption, and hormone replacement therapy.

^b^ Models adjusted for age, centre, education, socioeconomic status, cigarette smoking status, family history of prostate cancer in first degree relatives, BMI, physical activity, alcohol consumption. Categories for missing values were created for family history of prostate cancer in first degree relatives and alcohol consumption

Supplemental Table 4. Associations of siesta and breast and prostate cancer risk adjusted for other habitual sleep characteristics, MCC-Spain, 2008–2013.

|  | Breast Cancer Cases  n=1,270 | Breast Cancer Controls  n=1,273 | OR^a^ | LCI | UCI | Prostate Cancer Cases  n=832 | Prostate Cancer Controls  n=963 | OR^b^ | LCI | UCI |
| --- | --- | --- | --- | --- | --- | --- | --- | --- | --- | --- |
| Siesta |  |  |  |  |  |  |  |  |  |  |
| Never | 616 | 669 | 1.00 | - | - | 257 | 332 | 1.00 | - | - |
| Ever | 654 | 604 | 1.22 | 1.03 | 1.43 | 575 | 631 | 1.22 | 0.98 | 1.51 |
| Frequency of Siesta (Days Per Week) |  |  |  |  |  |  |  |  |  |  |
| Never | 616 | 669 | 1.00 | - | - | 257 | 332 | 1.00 | - | - |
| <3 | 144 | 132 | 1.08 | 0.82 | 1.43 | 95 | 82 | 1.28 | 0.89 | 1.85 |
| 3-5 | 103 | 80 | 1.40 | 1.01 | 1.94 | 54 | 59 | 1.34 | 0.87 | 2.07 |
| 6-7 | 407 | 392 | 1.23 | 1.02 | 1.48 | 426 | 490 | 1.19 | 0.95 | 1.49 |
| Duration of Siesta (Minutes) |  |  |  |  |  |  |  |  |  |  |
| Never | 616 | 669 | 1.00 | - | - | 257 | 332 | 1.00 | - | - |
| <15 | 95 | 106 | 1.07 | 0.78 | 1.46 | 62 | 74 | 1.07 | 0.72 | 1.60 |
| 15-29 | 140 | 149 | 1.06 | 0.81 | 1.38 | 127 | 130 | 1.27 | 0.93 | 1.74 |
| 30-59 | 196 | 176 | 1.18 | 0.93 | 1.51 | 162 | 175 | 1.25 | 0.94 | 1.67 |
| 60+ | 223 | 173 | 1.49 | 1.17 | 1.89 | 224 | 252 | 1.21 | 0.93 | 1.57 |

Note the sum does not equal the total due to missing data.

^a^ Models adjusted for age, centre, education, socioeconomic status, cigarette smoking status, family history of breast cancer in first degree relatives, BMI, physical activity, alcohol consumption, parity, age at first child, oral contraceptive, hormone replacement therapy, age at menarche, menopausal status, sleep duration, ever sleep problems, timing of sleep. Categories for missing values were created for family history of breast cancer in first degree relatives, alcohol consumption, and hormone replacement therapy.

^b^ Models adjusted for age, centre, education, socioeconomic status, cigarette smoking status, family history of prostate cancer in first degree relatives, BMI, physical activity, alcohol consumption, sleep duration, ever sleep problems, timing of sleep.

Categories for missing values were created for family history of prostate cancer in first degree relatives and alcohol consumption.

Supplemental Table 5. Associations of siesta and other habitual sleep characteristics and breast and prostate cancer risk, MCC-Spain, 2008–2013.

|  | Breast Cancer Cases  n=1,543 | Breast Cancer Controls  n=1,560 | OR^a^ | LCI | UCI | *p* value | Prostate Cancer Cases  n=1,008 | Prostate Cancer Controls  n=1,150 | OR^b^ | LCI | UCI | *p* value |
| --- | --- | --- | --- | --- | --- | --- | --- | --- | --- | --- | --- | --- |
| Sleep Duration (h) |  |  |  |  |  | *0.05* |  |  |  |  |  | *0.47* |
| <7 |  |  |  |  |  |  |  |  |  |  |  |  |
| Never Siesta | 252 | 277 | 1.00 | - | - |  | 117 | 125 | 1.00 | - | - |  |
| Ever Siesta | 216 | 233 | 1.00 | 0.76 | 1.31 |  | 225 | 257 | 0.93 | 0.67 | 1.30 |  |
| 7 |  |  |  |  |  |  |  |  |  |  |  |  |
| Never Siesta | 241 | 250 | 1.00 | - | - |  | 85 | 116 | 1.00 | - | - |  |
| Ever Siesta | 242 | 229 | 1.14 | 0.87 | 1.50 |  | 203 | 231 | 1.17 | 0.81 | 1.70 |  |
| 7+ |  |  |  |  |  |  |  |  |  |  |  |  |
| Never Siesta | 268 | 317 | 1.00 | - | - |  | 114 | 140 | 1.00 | - | - |  |
| Ever Siesta | 324 | 254 | 1.49 | 1.17 | 1.91 |  | 264 | 281 | 1.24 | 0.89 | 1.73 |  |
| Ever Sleep Problems |  |  |  |  |  | *0.42* |  |  |  |  |  | *0.87* |
| No |  |  |  |  |  |  |  |  |  |  |  |  |
| Never Siesta | 453 | 491 | 1.00 | - | - |  | 245 | 287 | 1.00 | - | - |  |
| Ever Siesta | 470 | 400 | 1.29 | 1.07 | 1.57 |  | 513 | 553 | 1.12 | 0.90 | 1.41 |  |
| Yes |  |  |  |  |  |  |  |  |  |  |  |  |
| Never Siesta | 308 | 353 | 1.00 | - | - |  | 71 | 94 | 1.00 | - | - |  |
| Ever Siesta | 312 | 316 | 1.10 | 0.87 | 1.39 |  | 179 | 216 | 1.19 | 0.79 | 1.80 |  |
| Timing of Sleep |  |  |  |  |  | *0.36* |  |  |  |  |  | *0.07* |
| Sleep Before 00h |  |  |  |  |  |  |  |  |  |  |  |  |
| Never Siesta | 306 | 309 | 1.00 | - | - |  | 150 | 184 | 1.00 | - | - |  |
| Ever Siesta | 321 | 302 | 1.15 | 0.91 | 1.46 |  | 324 | 293 | 1.40 | 1.04 | 1.87 |  |
| Sleep After 00h |  |  |  |  |  |  |  |  |  |  |  |  |
| Never Siesta | 455 | 535 | 1.00 | - | - |  | 166 | 197 | 1.00 | - | - |  |
| Ever Siesta | 461 | 414 | 1.31 | 1.08 | 1.59 |  | 368 | 476 | 0.93 | 0.72 | 1.24 |  |

Note the sum does not equal the total due to missing data.

^a^ Models adjusted for age, centre, education, socioeconomic status, cigarette smoking status, family history of breast cancer in first degree relatives, BMI, physical activity, alcohol consumption, parity, age at first child, oral contraceptive, hormone replacement therapy, age at menarche, menopausal status. Categories for missing values were created for family history of breast cancer in first degree relatives, alcohol consumption, and hormone replacement therapy.

^b^ Models adjusted for age, centre, education, socioeconomic status, cigarette smoking status, family history of prostate cancer in first degree relatives, BMI, physical activity, alcohol consumption. Categories for missing values were created for family history of prostate cancer in first degree relatives and alcohol consumption.

Supplemental Table 6. Associations of selected habitual sleep characteristics and breast cancer risk by menopausal status, MCC-Spain, 2008–2013.

|  | Premenopausal Cases  n=561 | Premenopausal Controls  n=489 | OR^a^ | LCI | UCI | Postmenopausal Cases  n=982 | Postmenopausal Controls  n=1,071 | OR^a^ | LCI | UCI | *p* value |
| --- | --- | --- | --- | --- | --- | --- | --- | --- | --- | --- | --- |
| Sleep Duration (h) |  |  |  |  |  |  |  |  |  |  | *0.93* |
| <7 | 132 | 125 | 0.96 | 0.69 | 1.34 | 336 | 385 | 0.95 | 0.75 | 1.20 |  |
| 7 | 218 | 197 | 1.00 | - | - | 265 | 282 | 1.00 | - | - |  |
| >7 | 211 | 167 | 1.14 | 0.85 | 1.54 | 381 | 404 | 0.98 | 0.78 | 1.23 |  |
| Ever Sleep Problems |  |  |  |  |  |  |  |  |  |  | *0.45* |
| No | 383 | 336 | 1.00 | - | - | 540 | 555 | 1.00 | - | - |  |
| Yes | 178 | 153 | 0.94 | 0.71 | 1.25 | 442 | 516 | 0.93 | 0.78 | 1.12 |  |
| Timing of Sleep |  |  |  |  |  |  |  |  |  |  | *0.81* |
| Sleep Before 00h | 228 | 195 | 1.00 | - | - | 399 | 416 | 1.00 | - | - |  |
| Sleep After 00h | 333 | 294 | 0.96 | 0.73 | 1.26 | 583 | 655 | 0.88 | 0.73 | 1.06 |  |
| Siesta |  |  |  |  |  |  |  |  |  |  | *0.75* |
| Never | 297 | 280 | 1.00 | - | - | 464 | 564 | 1.00 | - | - |  |
| Ever | 264 | 209 | 1.17 | 0.90 | 1.52 | 518 | 507 | 1.26 | 1.05 | 1.51 |  |

Note the sum does not equal the total due to missing data.

^a^ Models adjusted for age, centre, education, socioeconomic status, cigarette smoking status, family history of breast cancer in first degree relatives, BMI, physical activity, alcohol consumption, parity, age at first child, oral contraceptive, age at menarche. Categories for missing values were created for family history of breast cancer in first degree relatives, and alcohol consumption.

Supplemental Table 7. Associations of various habitual sleep characteristics and breast cancer risk by night shift work history and chronotype, MCC-Spain, 2008–2013.

|  | Never night shift | | | | | Ever night shift | | | | | Always housewife | | | | |  |
| --- | --- | --- | --- | --- | --- | --- | --- | --- | --- | --- | --- | --- | --- | --- | --- | --- |
|  | Cases  n=1,175 | Controls  n=1,129 | OR^a^ | LCI | UCI | Cases  n=205 | Controls  n=176 | OR^a^ | LCI | UCI | Cases  n=132 | Controls  n=209 | OR^a^ | LCI | UCI | *p* value |
| Sleep Duration (h) |  |  |  |  |  |  |  |  |  |  |  |  |  |  |  | *0.95* |
| <7 | 345 | 357 | 0.95 | 0.76 | 1.18 | 67 | 64 | 1.07 | 0.61 | 1.88 | 47 | 73 | 0.72 | 0.33 | 1.55 |  |
| 7 | 386 | 375 | 1.00 | - | - | 62 | 56 | 1.00 | - | - | 24 | 38 | 1.00 | - | - |  |
| >7 | 444 | 397 | 1.06 | 0.86 | 1.30 | 76 | 56 | 1.18 | 0.68 | 1.05 | 61 | 98 | 0.75 | 0.36 | 1.58 |  |
| Ever Sleep Problems |  |  |  |  |  |  |  |  |  |  |  |  |  |  |  | *0.47* |
| No | 726 | 653 | 1.00 | - | - | 115 | 100 | 1.00 | - | - | 69 | 111 | 1.00 | - | - |  |
| Yes | 449 | 476 | 0.89 | 0.74 | 1.06 | 90 | 76 | 1.18 | 0.74 | 1.89 | 63 | 98 | 1.16 | 0.69 | 1.96 |  |
| Timing of Sleep |  |  |  |  |  |  |  |  |  |  |  |  |  |  |  | *0.65* |
| Sleep Before 00h | 491 | 459 | 1.00 | - | - | 80 | 59 | 1.00 | - | - | 46 | 73 | 1.00 | - | - |  |
| Sleep After 00h | 684 | 670 | 0.90 | 0.76 | 1.08 | 125 | 117 | 0.85 | 0.53 | 1.37 | 86 | 136 | 0.79 | 0.46 | 1.34 |  |
| Siesta |  |  |  |  |  |  |  |  |  |  |  |  |  |  |  | *0.79* |
| Never | 582 | 624 | 1.00 | - | - | 98 | 86 | 1.00 | - | - | 67 | 109 | 1.00 | - | - |  |
| Ever | 593 | 505 | 1.26 | 1.06 | 1.49 | 107 | 90 | 1.15 | 0.73 | 1.81 | 65 | 100 | 1.13 | 0.68 | 1.86 |  |
|  | Morning Chronotype | | | | | Neither Chronotype | | | | | Evening Chronotype | | | | |  |
|  | Cases  n=458 | Controls  n=504 | OR^a^ | LCI | UCI | Cases  n=494 | Controls  n=504 | OR^a^ | LCI | UCI | Cases  n=203 | Controls  n=282 | OR^a^ | LCI | UCI | *p* value |
| Sleep Duration (h) |  |  |  |  |  |  |  |  |  |  |  |  |  |  |  | *0.48* |
| <7 | 148 | 173 | 0.92 | 0.67 | 1.28 | 126 | 169 | 0.74 | 0.52 | 1.06 | 89 | 84 | 1.00 | 0.63 | 1.60 |  |
| 7 | 160 | 164 | 1.00 | - | - | 165 | 156 | 1.00 | - | - | 92 | 90 | 1.00 | - | - |  |
| >7 | 150 | 167 | 0.97 | 0.70 | 1.35 | 203 | 179 | 1.03 | 0.74 | 1.42 | 122 | 108 | 1.14 | 0.73 | 1.78 |  |
| Ever Sleep Problems |  |  |  |  |  |  |  |  |  |  |  |  |  |  |  | *0.99* |
| No | 283 | 297 | 1.00 | - | - | 298 | 284 | 1.00 | - | - | 183 | 159 | 1.00 | - | - |  |
| Yes | 175 | 207 | 0.92 | 0.70 | 1.22 | 196 | 220 | 0.94 | 0.71 | 1.24 | 120 | 123 | 0.87 | 0.60 | 1.27 |  |
| Timing of Sleep |  |  |  |  |  |  |  |  |  |  |  |  |  |  |  | *0.79* |
| Sleep Before 00h | 269 | 289 | 1.00 | - | - | 175 | 160 | 1.00 | - | - | 61 | 51 | 1.00 | - | - |  |
| Sleep After 00h | 189 | 215 | 0.93 | 0.71 | 1.23 | 319 | 344 | 0.74 | 0.56 | 1.00 | 242 | 231 | 0.92 | 0.58 | 1.45 |  |
| Siesta |  |  |  |  |  |  |  |  |  |  |  |  |  |  |  | *0.44* |
| Never | 243 | 269 | 1.00 | - | - | 233 | 275 | 1.00 | - | - | 148 | 159 | 1.00 | - | - |  |
| Ever | 215 | 235 | 1.00 | 0.76 | 1.31 | 261 | 229 | 1.33 | 1.02 | 1.75 | 155 | 123 | 1.45 | 1.00 | 2.08 |  |

Note the sum does not equal the total due to missing data.

^a^ Models adjusted for age, centre, education, socioeconomic status, cigarette smoking status, family history of breast cancer in first degree relatives, BMI, physical activity, alcohol consumption, parity, age at first child, oral contraceptive, hormone replacement therapy, age at menarche, menopausal status. Categories for missing values were created for family history of breast cancer in first degree relatives, alcohol consumption, and hormone replacement therapy.

Supplemental Table 8. Associations of various habitual sleep characteristics and prostate cancer risk by night shift work history and chronotype, MCC-Spain, 2008–2013.

|  | Never night shift | | | | | Ever night shift | | | | |  | | | | |  |
| --- | --- | --- | --- | --- | --- | --- | --- | --- | --- | --- | --- | --- | --- | --- | --- | --- |
|  | Cases  n=710 | Controls  n=851 | OR^a^ | LCI | UCI | Cases  n=296 | Controls  n=297 | OR^a^ | LCI | UCI |  |  |  |  |  | *p* value |
| Sleep Duration (h) |  |  |  |  |  |  |  |  |  |  |  |  |  |  |  | *0.30* |
| <7 | 237 | 292 | 0.97 | 0.75 | 1.26 | 105 | 90 | 1.28 | 0.81 | 2.04 |  |  |  |  |  |  |
| 7 | 213 | 265 | 1.00 | - | - | 75 | 81 | 1.00 | - | - |  |  |  |  |  |  |
| >7 | 260 | 294 | 1.19 | 0.91 | 1.54 | 116 | 126 | 1.05 | 0.67 | 1.65 |  |  |  |  |  |  |
| Ever Sleep Problems |  |  |  |  |  |  |  |  |  |  |  |  |  |  |  | *0.35* |
| No | 534 | 627 | 1.00 | - | - | 223 | 211 | 1.00 | - | - |  |  |  |  |  |  |
| Yes | 176 | 224 | 0.94 | 0.74 | 1.20 | 73 | 86 | 0.77 | 0.51 | 1.15 |  |  |  |  |  |  |
| Timing of Sleep |  |  |  |  |  |  |  |  |  |  |  |  |  |  |  | *0.05* |
| Sleep Before 00h | 346 | 344 | 1.00 | - | - | 126 | 131 | 1.00 | - | - |  |  |  |  |  |  |
| Sleep After 00h | 364 | 507 | 0.70 | 0.57 | 0.87 | 170 | 166 | 1.13 | 0.78 | 1.63 |  |  |  |  |  |  |
| Siesta |  |  |  |  |  |  |  |  |  |  |  |  |  |  |  | *0.42* |
| Never | 215 | 273 | 1.00 | - | - | 100 | 106 | 1.00 | - | - |  |  |  |  |  |  |
| Ever | 495 | 578 | 1.13 | 0.90 | 1.42 | 196 | 191 | 1.10 | 0.75 | 1.62 |  |  |  |  |  |  |
|  | Morning Chronotype | | | | | Neither Chronotype | | | | | Evening Chronotype | | | | |  |
|  | Cases  n=426 | Controls  n=460 | OR^a^ | LCI | UCI | Cases  n=280 | Controls  n=328 | OR^a^ | LCI | UCI | Cases  n=110 | Controls  n=117 | OR^a^ | LCI | UCI | *p* value |
| Sleep Duration (h) |  |  |  |  |  |  |  |  |  |  |  |  |  |  |  | *0.39* |
| <7 | 153 | 167 | 1.15 | 0.81 | 1.62 | 91 | 90 | 1.20 | 0.78 | 1.85 | 39 | 33 | 1.47 | 0.69 | 3.11 |  |
| 7 | 122 | 144 | 1.00 | - | - | 89 | 106 | 1.00 | - | - | 39 | 43 | 1.00 | - | - |  |
| >7 | 151 | 149 | 1.29 | 0.90 | 1.84 | 100 | 132 | 0.85 | 0.56 | 1.29 | 32 | 41 | 0.80 | 0.38 | 1.71 |  |
| Ever Sleep Problems |  |  |  |  |  |  |  |  |  |  |  |  |  |  |  | *0.19* |
| No | 327 | 341 | 1.00 | - | - | 213 | 247 | 1.00 | - | - | 72 | 87 | 1.00 | - | - |  |
| Yes | 99 | 119 | 0.91 | 0.66 | 1.27 | 67 | 81 | 0.99 | 0.66 | 1.48 | 38 | 30 | 1.54 | 0.79 | 2.98 |  |
| Timing of Sleep |  |  |  |  |  |  |  |  |  |  |  |  |  |  |  | *0.53* |
| Sleep Before 00h | 261 | 258 | 1.00 | - | - | 93 | 96 | 1.00 | - | - | 21 | 14 | 1.00 | - | - |  |
| Sleep After 00h | 165 | 202 | 0.81 | 0.61 | 1.08 | 187 | 232 | 0.78 | 0.54 | 1.14 | 89 | 103 | 0.54 | 0.24 | 1.25 |  |
| Siesta |  |  |  |  |  |  |  |  |  |  |  |  |  |  |  | *0.35* |
| Never | 135 | 158 | 1.00 | - | - | 78 | 97 | 1.00 | - | - | 45 | 36 | 1.00 | - | - |  |
| Ever | 291 | 302 | 1.23 | 0.91 | 1.67 | 202 | 231 | 1.11 | 0.76 | 1.64 | 65 | 81 | 0.76 | 0.41 | 1.42 |  |

Note the sum does not equal the total due to missing data.

^a^ Models adjusted for age, centre, education, socioeconomic status, cigarette smoking status, family history of breast cancer in first degree relatives, BMI, physical activity, alcohol consumption. Categories for missing values were created for family history of prostate cancer in first degree relatives and alcohol consumption.

Supplemental Table 9. Associations of various sleep characteristics at around age 40 years and breast and prostate cancer risk, MCC-Spain, 2008–2013.

|  | Around age 40 years | | | | | Around age 40 years | | | | |
| --- | --- | --- | --- | --- | --- | --- | --- | --- | --- | --- |
|  | Breast Cancer Cases  n=1,052 | Breast Cancer Controls  n=1,093 | OR^a^ | LCI | UCI | Prostate Cancer Cases  n=751 | Prostate Cancer Controls  n=827 | OR^b^ | LCI | UCI |
| Sleep Duration^c^ Weekdays (h) |  |  |  |  |  |  |  |  |  |  |
| <6 | 59 | 64 | 0.82 | 0.55 | 1.22 | 65 | 70 | 1.07 | 0.72 | 1.58 |
| 6 | 157 | 183 | 0.76 | 0.58 | 0.99 | 152 | 171 | 1.01 | 0.76 | 1.34 |
| 7 | 371 | 339 | 1.00 | - | - | 291 | 332 | 1.00 | - | - |
| 8 | 333 | 366 | 0.86 | 0.69 | 1.06 | 192 | 195 | 1.22 | 0.93 | 1.6 |
| 9+ | 131 | 141 | 0.87 | 0.65 | 1.17 | 50 | 57 | 1.05 | 0.68 | 1.64 |
| Per 1 hour |  |  | 1.03 | 0.95 | 1.11 |  |  | 1.01 | 0.93 | 1.09 |
| Sleep Duration^c^ Weekends (h) |  |  |  |  |  |  |  |  |  |  |
| <6 | 23 | 19 | 1.28 | 0.66 | 2.49 | 14 | 20 | 0.80 | 0.38 | 1.69 |
| 6 | 46 | 53 | 0.90 | 0.56 | 1.43 | 48 | 53 | 1.07 | 0.66 | 1.71 |
| 7 | 156 | 165 | 1.00 | - | - | 129 | 148 | 1.00 | - | - |
| 8 | 337 | 376 | 0.92 | 0.7 | 1.21 | 210 | 238 | 1.07 | 0.78 | 1.47 |
| 9+ | 490 | 480 | 1.07 | 0.81 | 1.4 | 350 | 368 | 1.14 | 0.85 | 1.54 |
| Per 1 hour |  |  | 1.02 | 0.94 | 1.09 |  |  | 1.07 | 0.99 | 1.16 |
| Timing of Sleep Weekdays |  |  |  |  |  |  |  |  |  |  |
| Sleep Before 23h | 135 | 132 | 1.00 | - | - | 214 | 198 | 1.00 | - | - |
| Sleep at 23-00h | 391 | 427 | 0.88 | 0.66 | 1.17 | 291 | 321 | 0.85 | 0.65 | 1.11 |
| Sleep at 00-01h | 483 | 486 | 0.96 | 0.72 | 1.27 | 216 | 260 | 0.84 | 0.63 | 1.13 |
| Sleep After 01h | 42 | 48 | 0.79 | 0.48 | 1.3 | 29 | 46 | 0.54 | 0.32 | 0.92 |
| Timing of Sleep Weekends |  |  |  |  |  |  |  |  |  |  |
| Sleep Before 23h | 30 | 42 | 1.00 | - | - | 82 | 51 | 1.00 | - | - |
| Sleep at 23-00h | 158 | 174 | 1.15 | 0.67 | 1.98 | 200 | 224 | 0.55 | 0.36 | 0.83 |
| Sleep at 00-01h | 719 | 744 | 1.20 | 0.72 | 1.99 | 416 | 470 | 0.57 | 0.38 | 0.85 |
| Sleep After 01h | 145 | 133 | 1.26 | 0.72 | 2.21 | 53 | 82 | 0.40 | 0.23 | 0.67 |
| Social Jetlag^d^ |  |  |  |  |  |  |  |  |  |  |
| 0-1h | 239 | 268 | 1.00 | - | - | 196 | 193 | 1.00 | - | - |
| 1-2h | 491 | 540 | 0.95 | 0.75 | 1.19 | 243 | 293 | 0.88 | 0.67 | 1.17 |
| 2+h | 309 | 275 | 1.14 | 0.88 | 1.49 | 299 | 324 | 0.89 | 0.68 | 1.18 |

Note the sum does not equal the total due to missing data

^a^ Models adjusted for age, centre, education, socioeconomic status, cigarette smoking status, family history of breast cancer in first degree relatives, BMI, physical activity, alcohol consumption, parity, age at first child, oral contraceptive, hormone replacement therapy, age at menarche, menopausal status. Categories for missing values were created for family history of breast cancer in first degree relatives, alcohol consumption, and hormone replacement therapy.

^b^ Models adjusted for age, centre, education, socioeconomic status, cigarette smoking status, family history of prostate cancer in first degree relatives, BMI, physical activity, alcohol consumption. Categories for missing values were created for family history of prostate cancer in first degree relatives and alcohol consumption.

^c^ Based on responses to questions for the time the participant usually turns off the lights to go to sleep and the time the participant usually wakes up.

^d^ The absolute difference in hours of midpoints of sleep between weekdays and weekends.
